# Ruthenium Decorated Tris-Silylated Germanium Zintl Clusters Featuring an Unexpected Ligand Arrangement

**DOI:** 10.3390/molecules30061247

**Published:** 2025-03-11

**Authors:** Nicole S. Willeit, Viktor Hlukhyy, Thomas F. Fässler

**Affiliations:** 1 Department of Chemistry, TUM School of Natural Sciences, Technical University of Munich (TUM), Lichtenbergstraße 4, D-85748 Garching, Germanyviktor.hlukhyy@lrz.tum.de (V.H.); 2 Wacker Institute of Silicon Chemistry, Technical University of Munich (TUM), Lichtenbergstraße 4, D-85748 Garching, Germany

**Keywords:** cluster compounds, germanium, ruthenium, *Zintl* cluster, counterion

## Abstract

The incorporation of transition metal atoms into [Ge_9_] clusters is a widely studied area of *Zintl*-cluster chemistry. Recently, it was shown that clusters comprising single transition metal atoms in the cluster surface show catalytic properties. Here, we present a synthetic approach to four new compounds comprising silylated Ge_9_ clusters with organometallic ruthenium complexes. [η^5^-Ge_9_Hyp_3_]RuCp* (**1**), [η^1^-Ge_9_(Si*^t^*Bu_2_H)_3_]RuCp(PPh_3_)_2_ (**2**), and [Hyp_3_Ge_9_][RuCp(PPh_3_)_2_(MeCN)] (**3b**) (Cp = cyclopentadienyl, Cp* = pentamethylcyclopentadienyl, Hyp = Si(SiMe_3_)_3_, Ph = C_6_H_5_, *^t^*Bu = tert-butyl) were characterized by means of NMR spectroscopy and single-crystal structure determination. In the case of **2**, a new isomer with an approximated *C*_4*v*_ symmetric monocapped square antiprism of nine Ge atoms with an unexpected ligand arrangement comprising three ditertbutylsilane ligands attached to the open square was obtained. [Hyp_3_Ge_9_][RuCp(PPh_3_)_2_] (**3a**) was characterized via NMR spectroscopy and LIFDI mass spectrometry. Overall, we were able to show that the steric demand of the ligands Cp vs. Cp* and hypersilylchloride vs. ditertbutylsilane strongly influence the arrangement of the atoms and ligands on the cluster. In addition, the solvent also affects the cluster, as it appears that the ruthenium atom in **3a** dissociates from the cluster surface upon acetonitrile coordination to form **3b**. These results show that choosing the right synthetic tools and ligands makes a big difference in the outcome of the metalation reaction.

## 1. Introduction

Nine atomic *Zintl*-clusters occur in the phases *A*_12_*E*_17_ (*A* = Na, K, Rb, Cs; *E* = Si, Ge, Sn) and *A*_4_*E*_9_ (*A* = Na, K, Rb, Cs; *E* = Ge, Sn, Pb), while in the case of *A*_12_*E*_17_ additionally, tetrahedral four atomic clusters exist. Considering follow-up chemistry, the phase with the nominal composition *A*_4_*E*_9_, which is available for Ge–Pb, is favored [1,2,3,4,5,6,7]. Among the nine atomic clusters, germanium clusters are the best studied, and many functionalization reactions are well established. The silylation of [Ge_9_]^4−^ units is thereby one of the most important reactions as it reduces the charge of the cluster and makes the cluster soluble in common organic solvents, such as acetonitrile or tetrahydrofuran. In contrast, the naked *Zintl* anion is only soluble in highly polar solvents, such as ethylenediamine, dimethylformamide, or liquid ammonia. Reactions in the latter solvents lead to a maximum of two substituents at the cluster, and reactions with organosilicon halides are impossible due to reactions of the silyl group with ethylenediamine [8,9,10]. However, through heterogenous reactions in acetonitrile or tetrahydrofuran, many different silyl groups have been attached to [Ge_9_]^4−^ to give K[R_3_Ge_9_] with R = Hyp, Si*^i^*Bu_3_, Si*^i^*Pr_3_, SiEt_3_, SiH*^t^*Bu_2_, and SiPh_2_R′ (R′ = -CH=CH_2_, -(CH_2_)_3_CH=CH_2_) [11,12,13]. Besides the addition of three identical substituents, mixed silylated cluster compounds are also known. The starting material K_2_[Hyp_2_Ge_9_] can be synthesized in a stoichiometric reaction of K_4_Ge_9_ and hypersilylchloride. Further reaction with another silylchloride leads to the mixed silylated species K[Hyp_2_(SiPh_2_R′)Ge_9_] (R′ = -CH=CH_2_, -(CH_2_)_3_CH=CH_2_) [12]. Uncharged clusters are obtained from the onefold negatively charged compound [Hyp_3_Ge_9_]^−^ by reaction with either organotin halides, acid chlorides, or halogenated hydrocarbons to form [R″Hyp_3_Ge_9_] with R″ = -CH_2_CH=CH_2_, -(CH_2_)_3_CH=CH_2_, -C(CH_3_)_3_, -COC_6_H_5_, -COCH_3_, -COCH(CH_3_)_2_, -Sn(C_6_H_5_)_3_, -CH_2_CH_3_, and -Sn({CH_2_}_3_CH_3_)_3_ [14,15,16,17]. Additionally, silylated cluster species can be reacted with metal complexes to gain metalated cluster species. Various bonding modes of these clusters with transition metal atoms are known.

**Figure 1:**
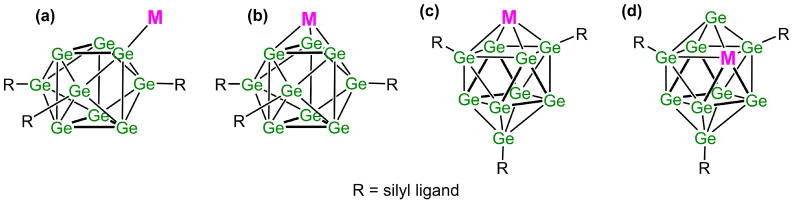
Different binding modes for the metal atom towards silylated cluster species: (**a**) single Ge–M bond (η^1^-coordination), (**b**) capping the triangular face (η^3^-coordination), (**c**) capping the square face (η^4^-coordination), and (**d**) occupying a vertex of the square (η^5^-coordination).

η^1^-coordination of the cluster comprising a single Ge–M bond is observed for [(η^1^-Ge_9_Hyp_3_)Cr(CO)_5_]^−^ (Figure 1a) [18]. Metal atoms capping triangular faces thus forming η^3^-coordination to the cluster (Figure 1b) are observed in (η^3^-Ge_9_Hyp_3_)*M*NHC^Dipp^ (*M* = Cu, Ag, Au) [19], (η^3^-Ge_9_Hyp_3_)*ML* (*M*/*L* = Ni/dppe, Zn/Cp*, Cu/P*^i^*Pr_3_, MIC, CAAC) (MIC = mesoionic carbene, CAAC = cyclic(alkyl)amino carbene) [20,21], and (η^3^-Ge_9_Hyp_3_)Rh*L* (*L* = PMe_3_, PPh_3_, IMe_4_) (IMe_4_ = 1,3,4,5-Tetramethylimidazol-2-ylidene) [22,23]. η^4^-coordination (Figure 1c) is observed in (η^4^-Ge_9_Hyp_3_)RhCOD (COD = 1,5-cyclooctadiene), in which the *D*_3*h*_ symmetric shape of [Ge_9_Hyp_3_]^−^ changes to a pseudo bicapped quadratic antiprism of the product with the transition metal capping one of the squares [22,24]. Further metal atoms occupying a vertex of one of the squares of a bicapped quadratic antiprism and germanium atoms capping the squares are possible (Figure 1d). Consequently, the metal atom possesses η^5^-coordination with the clusters atoms. This structural motif is found in: (η^5^-Ge_9_Hyp_3_)Rhdppe (dppe = 1,2-bis(diphenylphosphino)ethane) [24], [(η^5^-Ge_9_Hyp_3_)*M*(CO)_3_]^−^ (*M* = Cr, Mo, W) [18,25], and (η^5^-Ge_9_Hyp_3_Et)NiP*R*_3_ (*R* = Ph, *^p^*tolyl, *^i^*Pr, Me) [26]. In addition, metal atoms can bridge two germanium cluster cores by coordinating to two triangular faces belonging to two different clusters forming dimers [(η^3^-Ge_9_Hyp_3_)*M*(η^3^-Ge_9_Hyp_3_)]^a−^ (*M*/a = Mn/0, Pd/2, Cu/1, Ag/1, Au/1, Zn/0, Cd/0, Hg/0) [27,28,29,30] as well as the oligomers [(η^3^-Ge_9_Hyp_3_)Cu{η^3^(η^3^-Ge_9_Hyp_3_)CuPPh_3_}] [29] and [{η^3^(η^3^-Ge_9_Hyp_3_)ZnTMS_3_}Zn{η^3^(η^3^-Ge_9_Hyp_3_)ZnTMS_3_}] [31]. In addition to the functionalization of the cluster core itself, the counterion potassium could also be varied, allowing the synthesis of [K-NHC^Dipp^_2_][Hyp_3_Ge_9_], as well as the complete exchange of the potassium ion in [NHC^Dipp^-H][Hyp_3_Ge_9_] [32]. In summary, these examples show that, on one hand, the field of germanium cluster chemistry is well developed but that, on the other hand, new aspects arise, such as the role of metalated germanium clusters as homogeneous catalysts for hydrogenation and isomerization reactions [22,23,24,26].

Herein, we report on further and novel synthetic approaches to ruthenium-decorated germanium *Zintl*-clusters and the influence of the solvent on the reaction product. Among others, [η^5^-Ge_9_Hyp_3_]RuCp* (**1**), [η^1^-Ge_9_(Si*^t^*Bu_2_H)_3_]RuCp(PPh_3_)_2_ (**2**), and [Hyp_3_Ge_9_][RuCp(PPh_3_)_2_(MeCN)] (**3b**) were characterized through single-crystal structure determination, showing the variation in the coordination modes of the Ru atom as well as the arrangement of the ligands on the cluster.

## 2. Results and Discussion

**Scheme 1:**
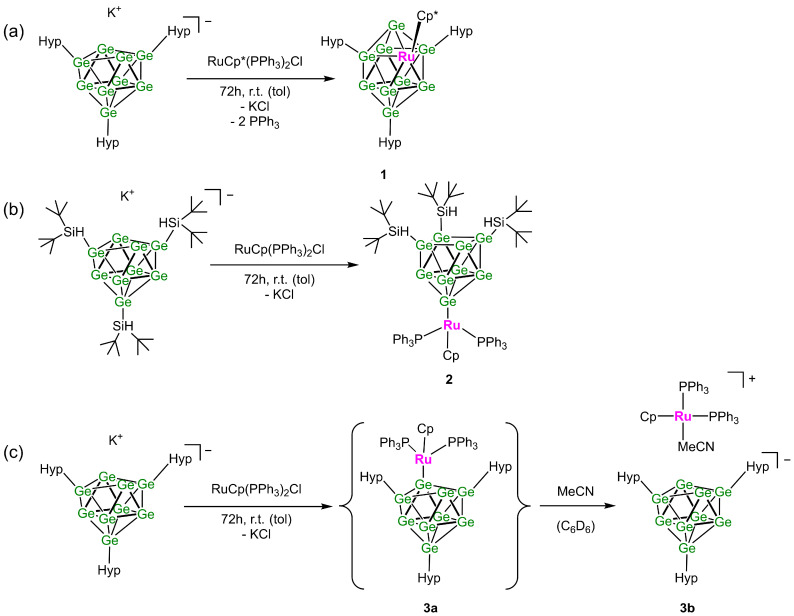
Different reactions occurring between silylated clusters and ruthenium complexes: (**a**) reaction between K[Hyp_3_Ge_9_] and RuCp*(PPh_3_)_2_Cl to give **1**, (**b**) reaction between K[(*^t^*Bu_2_HSi)_3_Ge_9_] and RuCp(PPh_3_)_2_Cl to give **2**, and (**c**) reaction between K[Hyp_3_Ge_9_] and RuCp(PPh_3_)_2_Cl to give the ruthenium decorated cluster **3a**, which reacts with acetonitrile to **3b**.

Reactions of the complexes RuCp*(PPh_3_)_2_Cl and RuCp(PPh_3_)_2_Cl with the silylated cluster species K[Hyp_3_Ge_9_] and K[(*^t^*Bu_2_HSi)_3_Ge_9_] resulted in various products in dependency on the steric demands of the silyl ligands Hyp and *^t^*Bu_2_HSi as well as of the ligands cyclopentadienyl versus pentamethylcyclopentadienyl that coordinate to the Ru atom (Scheme 1).

**Figure 2:**
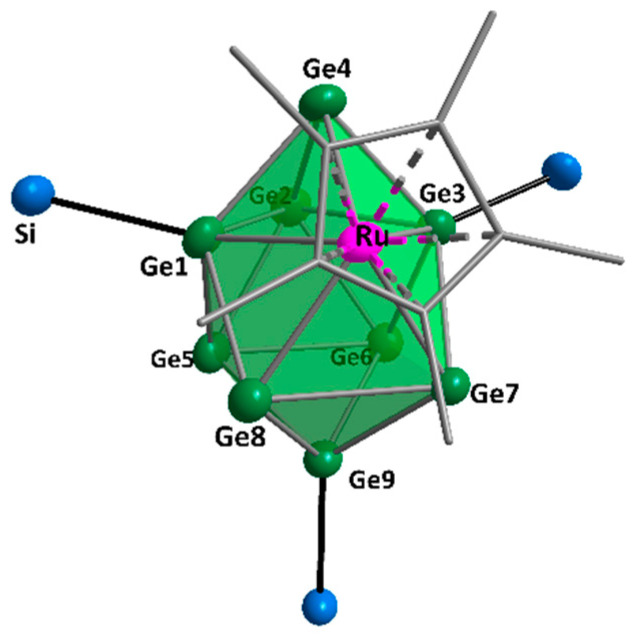
Molecular structure of [η^5^-Ge_9_Hyp_3_]RuCp* (**1**). All displacement ellipsoids are shown at a probability level of 50%. The [Ge_9_Ru] cluster core is shown as a green polyhedron. Ge, Si, Ru, and C atoms are depicted in green, dark blue, pink, and gray color, respectively. The TMS groups of the hypersilyl substituents are omitted for clarity and the Cp* ligand is shown in wire and frame mode for simplicity reasons. All hydrogen atoms are omitted for clarity.

Reaction of [Hyp_3_Ge_9_]^−^ comprising the sterically more demanding silyl ligand Hyp with RuCp*(PPh_3_)_2_Cl possessing another sterically demanding ligand Cp* leads to [η^5^-Ge_9_Hyp_3_]RuCp* (**1**) (Scheme 1a). The structure of the cluster was resolved by single-crystal X-ray diffraction, revealing a ten-atom bicapped square antiprismatic [Ge_9_Ru] cluster core, with ruthenium occupying one vertex of one square. In total, four ligands are bound to the cluster, three silyl ligands are bound to Ge1, Ge3, and Ge9, and the Cp* ligand is bound to Ru (Figure 2). The cluster core, thus, adopts a *C_s_*-symmetric distorted bicapped square antiprism. The Ge–Ge bond distances are in the range of 2.500(1)–3.188(2) Å, with Ge5–Ge9 being the shortest and Ge5–Ge8 being the longest distance. The bond distances in **1** are in good agreement with metalated cluster species known from the literature with metal atoms occupying the same position, such as (η^5^-Ge_9_Hyp_3_Et)*M*P*R*_3_ (*M*/*R* = Ni/PPh_3_, P*^p^*tolyl_3_, P*^i^*Pr_3_, PMe_3_; Pd/PPh_3_; Pt/PPh_3_) [26,33,34], (η^4^-Ge_9_Hyp_3_)RhCOD [18,25], and [(η^5^-Ge_9_Hyp_3_)*M*(CO)_3_]^−^ (*M* = Mo, W, Cr) [24], with Ge–Ge distances in the ranges 2.455–2.885 Å, 2.498–3.152 Å, and 2.502(5)–2.824(3) Å, respectively. In addition, the Ru atom has an overall η^10^-coordination (5 Ge atoms and 5 C atoms), with Ge–Ru bond lengths in the range of 2.579(1)–2.677(1) Å.

The composition of **1** in solution is confirmed by LIFDI mass spectrometry, which shows the mass peak of Hyp_3_[Ge_9_Ru]Cp* (Figure S8, Supplementary Materials). The structure of **1** is verified in solution as two signals for the hypersilyl ligands appear in the ^1^H-NMR spectra with a ratio of 2:1 (0.37 ppm and 0.77 ppm) in addition to the signals for the Cp* ligand at 1.71 ppm and two signals for the phenyl rings of the triphenylphosphine in the range of 7–7.5 ppm (Figure S3, Supplementary Materials). These results are corroborated by the ^13^C NMR spectrum, in which two chemically inequivalent silyl groups are detected at 3.4 ppm and 4.4 ppm, one signal for the Cp* ligand at 14.6 ppm, and two signals for the phenyl rings in the range of 128–135 ppm (Figure S6, Supplementary Materials). The ^29^Si NMR spectrum further confirms the existence of two chemically inequivalent silyl ligands (Figure S5, Supplementary Materials). According to the ^31^P NMR spectrum (Figure S4, Supplementary Materials), the PPh_3_ ligands are cleaved during the reaction, showing signals at −5.4 ppm for non-coordinating PPh_3_ as well as small amounts of residual RuCp*(PPh_3_)_2_Cl (40.8 ppm). In addition, a signal of small intensity is observed at 24.8 ppm, which could originate from PPh_3_O [35].

**Figure 3:**
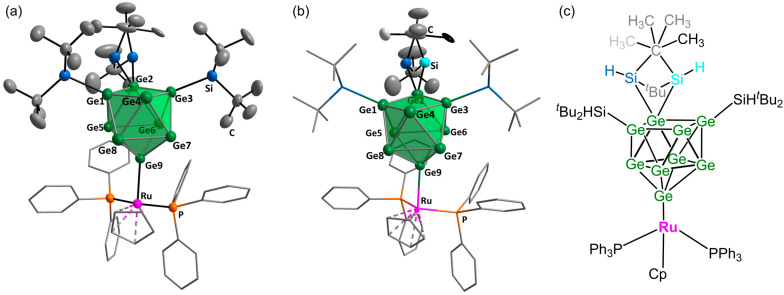
Molecular structure of (**a**) [η^1^-Ge_9_(Si*^t^*Bu_2_H)_3_]RuCp(PPh_3_)_2_ (**2**), (**b**) **2** with colored disordered silyl ligand, and (**c**) schematic drawing of the cluster and the disorder. All displacement ellipsoids are shown at a probability level of 50%. The core of the [Ge_9_] cluster is shown as a green polyhedron. Ge, Si, Ru, P, and C atoms are depicted in green, dark/light blue, pink, orange, and gray/black colors, respectively. The Si and C atoms of the disordered silyl ligand are colored as follows: dark blue Si and black C, occupancy 63.4(8)%; light blue Si and light gray C, occupancy 36.6(8)%; dark gray C, full occupancy. For (**a**), the Cp ligand and the phenyl rings of PPh_3_, and for (**b**), all ligands despite the silyl group bound to Ge2, are shown in wire and frame mode for simplicity reasons. All hydrogen atoms are omitted for clarity.

Reaction of [(*^t^*Bu_2_HSi)_3_Ge_9_]^−^ containing the sterically less demanding *^t^*Bu_2_HSi ligand with RuCp(PPh_3_)_2_Cl comprising another sterically less demanding ligand Cp (Scheme 1b) in toluene leads to the isolation of single crystals suitable for single-crystal structure determination through recrystallisation of the product from diethylether solution at −32 °C. The resulting product [η^1^-Ge_9_(Si*^t^*Bu_2_H)_3_]RuCp(PPh_3_)_2_ (**2**) with the overall symmetry *C*_1_ shows a threefold silylated cluster with the RuCp(PPh_3_)_2_ fragment bound to one Ge cluster atom forming a Ge–Ru bond (Figure 3). The three silyl groups are each bound to three neighboring germanium atoms (Ge1, Ge2, and Ge3), while the ruthenium atom is bound to Ge9. The occurrence of this unusual position of the silyl groups differs from that of known species, in which the Ge atoms to which silyl groups are attached are separated by ligand-free Ge cluster atoms. This observed ligand arrangement is quite surprising and marks a new isomer concerning fourfold substituted cluster species. The bond distances in **2** in the range of 2.460(2)–3.068(2) Å are in good agreement with those of **1** above, but here, Ge1–Ge2 is the shortest and Ge6–Ge7 is the longest bond. Compared to [K-2.2.2-crypt]_2_K_4_[(η^1^-Si_9_)W(CO)_4_] and [(η^1^-Ge_9_Hyp_3_)Cr(CO)_5_]^−^ with a W–Si *exo*-bond of 2.6649(13) Å and a Ge–Cr *exo*-bond of 2.553(1) Å [18,36], the Ru–Ge *exo*-bond of 2.4822(9) Å is slightly shorter. However, this is in good agreement with Ge–Ru bond distances reported in the literature [37,38,39]. Overall, Ru has a η^8^-coordination to one Ge atom, two P atoms, and five C atoms. Due to the *exo*-bonds of the three neighboring atoms Ge1, Ge2, and Ge3 to the three silyl groups, the Ge–Ge cluster bonds between these atoms are shortened with values for Ge1–Ge2 and Ge2–Ge3 of 2.460(2) Å and 2.477(2) Å, respectively. Such shortening of cluster bonds, which are regarded as delocalized bonds of the electron-deficient Ge_9_ Wade-clusters, can be interpreted as a tendency to form localized covalent two-center-two-electron bonds. Similar trends are observed for [Hyp_3_EtGe_9_], [Hyp_3_Ge_9_(CH_2_)_3_CH=CH_2_], [Hyp_3_Ge_9_(CO)Ph], and [Hyp_3_Ge_9_Sn*^n^*Bu_3_] with shortened Ge–Ge bonds in the range of 2.4428(8)–2.490(3) Å [14,15,17]. The silyl ligand bound to Ge2 is disordered; thus, the silicon atom is split to two positions with occupancies of 63.4(8)% and 36.6(8)% (Figure 3b,c, colored in dark and light blue, respectively). Accordingly, one of the two *^t^*Bu groups connected to these silicon atoms is disordered as well, as one C atom is split into two positions (Figure 3b,c, colored in black and light gray) with the same occupancies found for silicon, while the other two C atoms and the second *^t^*Bu group are not disordered and are, therefore, fully occupied (Figure 3b,c, colored in dark gray). The H atom directly bound to silicon of this silyl group could not be refined due to the disorder. Concerning the cluster type of **2**, a correspondence to a *C*_4*v*_ unit is more likely than *D*_3*h*_ as one height of an assumed trigonal prism is strongly elongated (3.700(2) Å versus 2.985(2) Å, 3.068(2) Å), and the ratio of the diagonals of the corresponding pseudo square face Ge1–Ge2–Ge3–Ge4 with 1.08 suggests a nearly quadratic face, which is nearly planar, as expressed by the torsion angle over the longest height of 3.93(5)° (Table 1). Similar trends and, therefore, a compliance with *C*_4*v*_ cluster types are observed for other fourfold substituted cluster species such as [Hyp_3_EtGe_9_], [Hyp_3_Ge_9_{(CH_2_)_3_CH=CH_2_}], [Hyp_3_Ge_9_(COPh)], and [(η^1^-Ge_9_Hyp_3_)Cr(CO)_5_]^−^ (Table 1) [14,15,17,18]. Notice, according to the electron count of Wade–Mingos [40,41] rules, all clusters possess 22 = 2 × 9 + 4 skeletal electrons (two electrons from each cluster atom by considering two electrons as a lone pair as well as four electrons due to the charge) and, thus, correspond to *nido*-clusters, in contrast to species [(Si*^t^*Bu_2_H)_3_Ge_9_]^−^ and (η^3^-Ge_9_Hyp_3_)ZnCp* with *D*_3*h*_ symmetry. The latter possesses almost equal prism heights, which, however, are strongly elongated, and three non-planar pseudo quadratic faces (Table 1) [13,28]. Such clusters with elongated heights are alternatives to *nido*-structures.

The ligand arrangement as observed in the crystal structure of **2** which approximates *C_S_* symmetry should lead to two signals for the silyl substituents in the NMR spectra, as observed for **1**. However, **2** is not stable in solution and decomposes rapidly according to NMR. Further characterization via NMR or LIFDI/MS was, therefore, not possible. However, elemental analysis of the crystalline product confirmed the formation of the product.

Reaction of [Hyp_3_Ge_9_]^−^ with the sterically more demanding Hyp groups and RuCp(PPh_3_)_2_Cl (Scheme 1c) forms according to the LIFDI mass spectrum (Figure S14, Supplementary Materials) Hyp_3_[Ge_9_Ru]Cp. The ^31^P NMR (Figure S10, Supplementary Materials) of the reaction product **3a** shows an intense new signal at 44.8 ppm beside one of low intensity of free phosphine at −5.4 ppm as well as one for residual RuCp(PPh_3_)_2_Cl at 39.5 ppm. The shift of the new signal is in the typical range of triphenylphosphine ligands of other metalated cluster species such as (η^5^-Ge_9_Hyp_3_Et)NiPPh_3_ (51.2 ppm) and (η^3^-Ge_9_Hyp_3_)RhPPh_3_ (49.3 ppm), all showing a low field shift compared to the unattached ligand [23,26,33]. These results hint at a RuCp(PPh_3_)_2_ fragment connected to the cluster. During the LIFDI measurement, the triphenylphosphine ligands may cleave off as is known for RuCp(PPh_3_)_2_Cl [42]. The ^1^H NMR (Figure S9, Supplementary Materials) shows one singlet for the three hypersilyl ligands at 0.53 ppm, a singlet for the protons of the cyclopentadienyl group at 4.73 ppm revealing the expected ratio 81:5, and a very broad signal for the phenyl rings of the phosphine at about 7 ppm. The ^13^C NMR (Figure S12, Supplementary Materials) also shows a singlet for the hypersilyl groups and a singlet for the cyclopentadienyl ligand. Again, the signals for the phenyl rings are not entirely clear as they partially overlap with the signal from benzene-d_6_. Temperature-dependent ^1^H NMR spectra (Figure S15, Supplementary Materials) show a 2:1 splitting of the signal of the hypersilyl ligands at low temperatures. Two sets of signals are also observed in the ^29^Si NMR for the silicon atoms directly bound to the cluster (−99.65 ppm and −78.67 ppm) and for the TMS groups at −12.44 ppm and −9.06 ppm, hinting towards chemically inequivalent silyl ligands. The results are in accordance with a Ru fragment connected to the cluster as observed for **2**, since otherwise only two instead of the observed four signals should occur in the ^29^Si NMR, and no splitting of the hypersilyl signal in the ^1^H NMR at low temperatures would occur. However, it was not possible to obtain single crystals of **3a,** and temperature-dependent measurements indicate a dynamic process structure. Such a dynamic process of a cluster with four ligands and three equivalent silyl ligands has been observed before for [Hyp_3_EtGe_9_] [17]. But, due to steric hindrance of the silyl ligands in the case of **3a**, no rearrangement of the silyl ligands as in **2** is expected. Therefore, Ru most likely forms a *exo*-bond to a Ge atom of a triangular face or is not connected to the clusters, as shown below for **3b**.

**Figure 4:**
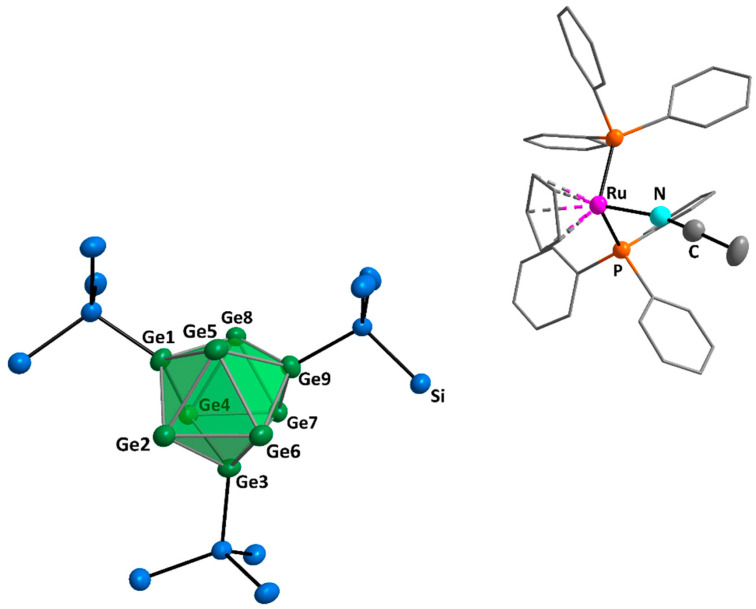
Molecular structure of [Hyp_3_Ge_9_][RuCp(PPh_3_)_2_(MeCN)] (**3b**). All displacement ellipsoids are shown at a probability level of 50%. The core of the [Ge_9_] cluster is shown as a green polyhedron. Ge, Si, Ru, P, N, and C atoms are depicted in green, dark blue, pink, orange, light blue, and gray colors, respectively. Methyl groups on the hypersilyl substituents are omitted for clarity. The Cp ligand and the phenyl rings of PPh_3_ are shown in wire and frame mode for simplicity reasons. All hydrogen atoms are omitted for clarity.

The intermediate product **3a** can be further reacted with acetonitrile in C_6_D_6_ (Scheme 1c). Thereby, the formation of about 25% of a new compound is indicated by a new singlet for the hypersilyl ligands in the ^1^H NMR spectrum at 0.65 ppm (Figure S16, Supplementary Materials) and a new signal in the ^31^P NMR spectrum at 42.4 ppm (Figure S17, Supplementary Materials). Heating **3a** in acetonitrile in a sealed NMR tube (Setup Figure S21, Supplementary Materials) gives a light reddish solution in addition to undissolved residues of **3a**. In the upper part of the NMR tube at lower temperature, crystals of **3b** suitable for single-crystal structure determination formed. Compound **3b** consists of a threefold silylated germanium cluster anion with overall *C*_1_ symmetry and a ruthenium complex counter ion [RuCp(PPh_3_)_2_(MeCN)]^+^. As in the presence of acetonitrile a change in the NMR spectra of **3a** is observed, we assume that the coordinated ruthenium fragment dissociates from the cluster upon coordinating to acetonitrile (Figure 4). Similarly, a compound of [Hyp_3_Ge_9_]^−^ with a more complex cation instead of a alkali metal cation was also found in a reaction of the anion with NHC (N-heterocyclic carbene) forming [Hyp_3_Ge_9_][NHC^Dipp^-H] [32]. The bond lengths within **3b** are in the range of 2.5213(5)–3.6934(4) Å, which is slightly longer than those reported for [Hyp_3_Ge_9_][NHC^Dipp^-H], with 2.5154(9)–2.5506(9) Å [32], but similar to those reported for [Hyp_3_Ge_9_][K-2.2.2.-crypt], with 2.5237(9)–3.5291(8) Å [8], as well as those observed for **1** and **2**. The shortest distance here is Ge1–Ge8 and the longest is Ge6–Ge7. The cluster in **3b** possesses *D*_3*h*_ symmetry and corresponds to an elongated tricapped trigonal prism, as also observed for K[(Si*^t^*Bu_2_H)_3_Ge_9_] and (η^3^-Ge_9_Hyp_3_)ZnCp* (Table 1) [13,28]. Here, again, Ru has an overall η^8^-coordination to two P atoms, five C atoms, and one N atom.

## 3. Materials and Methods

### 3.1. General

All manipulations were performed under a purified argon atmosphere using standard Schlenk and glovebox techniques. The solvents acetonitrile, hexane, toluene, and diethylether were dried over molecular sieves using the solvent purification system MB-SPS. All deuterated solvents were purchased from Sigma-Aldrich Taufkirchen, Germany and stored over molecular sieves (3 Å). The *Zintl* phase K_4_Ge_9_ was synthesized by heating a stoichiometric mixture of the elements K (Merck Darmstadt, Germany 99.8%) and Ge (Chempur Karlsruhe, Germany, 99.999%) at 650 °C for 46 h in a stainless steel autoclave [6]. Hypersilylchloride (Sigma-Aldrich Taufkirchen, Germany, 97%), di-*tert*-butylchlorosilane (Sigma-Aldrich Taufkirchen, Germany, 97%), chloro-(pentamethylcyclopentadienyl)-bis(triphenylphosphine)-ruthenium(II) (Sigma-Aldrich Taufkirchen, Germany, 98%), and chlorocyclopentadienylbis(triphenylphosphine)-ruthenium(II) (Sigma-Aldrich Taufkirchen, Germany, 98%) were used as received.

### 3.2. NMR Spectroscopy

All NMR spectra were recorded on a Bruker Ettlingen, Germany Avance Ultrashield 400 MHz. The signals of the ^1^H and ^13^C spectra were referenced to the signals of the used deuterated solvents benzene-*d*_6_ (7.16 ppm; 128.06 ppm), acetonitrile-*d*_3_ (1.94 ppm; 1.32 ppm, 118.26 ppm), and toluene-*d*_8_ (2.08 ppm, 6.97 ppm, 7.01 ppm, 7.09 ppm; 137.48 ppm, 128.87 ppm, 127.96 ppm, 125.13 ppm, 20.43 ppm) [43]. Chemical shifts are given in *d* values in parts per million (ppm). The coupling constants *J* are stated in Hz. Signal multiplicities are abbreviated as follows: s—singlet and m—multiplet. The spectra were evaluated with the program MestReNova [44].

### 3.3. Single-Crystal Structure Determination

A few crystals were transferred from the mother liquor into perfluoropolyalkylether under a cold N_2_ gas stream for single-crystal data collection. A single crystal was fixed on a glass fiber and positioned in a 150 K cold N_2_ gas stream. Single-crystal data collection was performed with an STOE Darmstadt, Germany Stadivari (Mo K_α_ radiation) diffractometer equipped with a DECTRIS Baden, Switzerland Pilatus 300 K detector by using the X-Area software package version 1.76.8.1 [45]. The crystal structures were solved by direct methods using *SHELX* software version 6.4.0 [46,47]. The positions of the hydrogen atoms were calculated and refined using a riding model. Unless otherwise stated, all non-hydrogen atoms were treated with anisotropic displacement parameters. For visualization, the crystal structures were plotted with *Diamond* version 3.2k[48].

### 3.4. LIFDI/MS

Liquid injection field desorption ionization mass spectrometry (LIFDI/MS) was measured directly from an inert atmosphere glovebox with a Thermo Fisher Scientific Munich, Germany Exactive Plus Orbitrap equipped with an ion source from Linden CMS [49].

### 3.5. Elemental Analysis

Elemental analysis was performed by the microanalytical laboratory at the Catalytic Research Centre of the Technical University of Munich. The elements C and H were determined by a combustion analyzer (elementar vario EL, Bruker Corp., Karlsruhe, Germany).

### 3.6. Syntheses

#### 3.6.1. Synthesis of K[Hyp_3_Ge_9_]

K[Hyp_3_Ge_9_] was synthesized according to a procedure in the literature [8]. K_4_Ge_9_ (7.31 g, 9.0 mmol) and hypersilylchloride (7.67 g, 27.1 mmol) were dissolved in 100 mL acetonitrile in a Schlenk tube. The resulting brown suspension is stirred at r.t. overnight and filtered over a Whatman Munich, Germany filter. Afterwards, the solvent is removed in vacuo, and the resulting residue is washed with hexane (6 × 20 mL). After drying in high vacuum, 10.14 g of K[Hyp_3_Ge_9_] (78%), were obtained as orange powder.

^1^H NMR (400 MHz, acetonitrile-*d*_3_, 298 K): 0.22 (s, 81H, TMS).

#### 3.6.2. Synthesis of K[(*^t^*Bu_2_HSi)_3_Ge_9_]

K[(*^t^*Bu_2_HSi)_3_Ge_9_] was synthesized according to a procedure from the literature [13]. K_4_Ge_9_ (1.00 g, 1.23 mmol) and *^t^*Bu_2_HSiCl (0.75 mL, 3.70 mmol) were dissolved in 15 mL acetonitrile in a Schlenk tube. The suspension turned brown after a few minutes and was stirred at room temperature for 72 h. After Whatman Munich, Germany filtration and washing with acetonitrile (2 × 5 mL), the solvent was removed in vacuo. Then, 1.00 g of K[(*^t^*Bu_2_HSi)_3_Ge_9_] (73%) were gained as a brown solid.

^1^H NMR (400 MHz, acetonitrile-*d*_3_, 298 K): 1.14 (s, 54H, C*H*_3_), 4.26 (s, 3H, Si*H*).

#### 3.6.3. Synthesis of [η^5^-Ge_9_Hyp_3_]RuCp* (**1**)

K[Hyp_3_Ge_9_] (1.00 g, 0.7 mmol) and RuCp*(PPh_3_)_2_Cl (0.55 g, 0.7 mmol) were weighted into a Schlenk tube, and 15 mL toluene were added. The resulting dark red solution was stirred at r.t. for 72 h. After Whatman Munich, Germany filtration and washing with toluene (5 mL), the solvent was removed in vacuo. Then, 0.98 g of Hyp_3_[Ge_9_Ru]Cp* + 2 PPh_3_ (corresponding to 0.74 g of **1**, 65%) were gained as a red–brown solid. For crystallization, **1** was dissolved in diethylether and stored at −32 °C. After 2 months, dark red block-shaped crystals, suitable for single-crystal X-ray diffraction, were obtained.

^1^H NMR (400 MHz, C_6_D_6_, 298 K): 0.37 (s, 54H, TMS), 0.77 (s, 27H, TMS), 1.71 (s, 15H, Cp*), 7.00–7.07 (m, 22H, Ph), 7.37–7.41 (m, 14H, Ph).

^13^C{^1^H} NMR (100 MHz, C_6_D_6_, 298 K): 3.36 (TMS), 4.36 (TMS), 14.58 (Cp), 128.83 (Ph), 134.19 (Ph).

^29^Si{^1^H} NMR (100 MHz, C_6_D_6_, 298 K): −101.40 (*Si*TMS_3_), −71.68 (*Si*TMS_3_), −10.18 (*Si*Me_3_), −9.06 (*Si*Me_3_).

LIFDI/MS: *m*/*z* = 1633.91 [Hyp_3_Ge_9_RuCp*]^+^, 1386.25 [Hyp_2_Ge_9_RuCp*]^+^.

Elemental analysis (**1** + 2 PPh_3_) calc: C 40.62 H 5.93, found: C 41.14 H 5.91.

#### 3.6.4. Synthesis of [η^1^-Ge_9_(Si*^t^*Bu_2_H)_3_]RuCp(PPh_3_)_2_ (**2**)

K[(*^t^*Bu_2_HSi)_3_Ge_9_] (100 mg, 0.09 mmol) and RuCp(PPh_3_)_2_Cl (65 mg, 0.09 mmol) were weighted into a Schlenk tube, and 5 mL toluene were added. The resulting dark red solution was stirred at r.t. for 72 h. After Whatman Munich, Germany filtration, the solvent was removed in vacuo and the residue was redissolved in 2 mL diethylether. This diethylether solution was stored at −32 °C for crystallization. After 2 months, dark red plate-shaped crystals, suitable for single-crystal X-ray diffraction, were obtained.

Elemental analysis (**2**) calc: C 44.00 H 5.23, found: C 43.83 H 5.17.

#### 3.6.5. Synthesis of [Hyp_3_Ge_9_][RuCp(PPh_3_)_2_] or [Hyp_3_Ge_9_][RuCp(PPh_3_)_2_(MeCN)] (**3a** and **3b**)

K[Hyp_3_Ge_9_] (1.00 g, 0.7 mmol) and RuCp(PPh_3_)_2_Cl (0.51 g, 0.7 mmol) were weighted into a Schlenk tube, and 15 mL toluene were added. The resulting dark red solution was stirred at r.t. for 72 h. After Whatman Munich, Germany filtration and washing with toluene (5 mL), the solvent was removed in vacuo. Then, 1.31 g of [Hyp_3_Ge_9_][RuCp(PPh_3_)_2_] (**3a**, 89%) were gained as a red–brown solid.

**3a** (0.03 g, 0.01 mmol) was further reacted with acetonitrile (3 μL, 0.04 mmol) in C_6_D_6_ to yield [Hyp_3_Ge_9_][RuCp(PPh_3_)_2_(MeCN)] (**3b**, 25%). For crystallization of **3b**, **3a** (0.07 g, 0.03 mmol) was sealed in an NMR tube with acetonitrile (1.5 mL). The lower part with undissolved **3a** was heated to 50 °C in an oil bath to dissolve parts of it, obtaining a colored solution, and it was mixed (Figure S21, Supplementary Materials). In the upper cooler part, orange–red block-shaped crystals of **3b,** suitable for single-crystal X-ray diffraction, were obtained after 3 weeks.

^1^H NMR (**3a**) (400 MHz, C_6_D_6_, 298 K): 0.53 (s, 81H, TMS), 4.73 (s, 5H, Cp), 6.89–7.08 (m, 36H, Ph).

^31^P{^1^H} NMR (**3a**) (162 MHz, C_6_D_6_, 298 K): 44.78 (PPh_3_@Ru).

^13^C{^1^H} NMR (**3a**) (100 MHz, C_6_D_6_, 298 K): 4.21 (TMS), 84.91 (Cp).

^29^Si{^1^H} NMR (**3a**) (100 MHz, C_6_D_6_, 298 K): −99.65 (*Si*TMS_3_), −78.67 (*Si*TMS_3_), −12.44 (*Si*Me_3_), −9.06 (*Si*Me_3_).

LIFDI/MS (**3a**)**:** *m*/*z* = 1562.77 [Hyp_3_Ge_9_RuCp]^+^.

Elemental analysis (**3a**) calc: C 39.13 H 5.60, found: C 39.99 H 5.67.

## 4. Conclusions

The formations of the three products **1**, **2**, and **3b**, which depend on the different steric demands of the reaction partners, hint at the following reaction paths. In compound **2,** a sterically less demanding Si*^t^*Bu_2_H ligand at the cluster allows for a η^1^ coordination of the RuCp(PPh_3_)_2_ fragment. This can be regarded as the first reaction step in any reaction of a cluster with a transition metal. The RuCp(PPh_3_)_2_ fragment forms a Ge–Ru bond, and, most interestingly, all three silyl groups are now connected to neighboring Ge atoms, forming an open square face of the polyhedron, allowing, most probably, the best optimization of the steric demands. In the presence of the sterically more demanding Hyp and Cp* instead of Si*^t^*Bu_2_H and Cp, respectively, the same entering step might occur; however, PPh_3_ ligands bound to the Ru atom dissociate and the Ru stabilizes the electron deficiency by entering the clusters sphere. Cp*Ru forms a cluster vertex, resulting in the formation of a [Ge_9_Ru] *closo*-cluster core. In the case of a sterically demanding cluster species and a less demanding ligand at the Ru atom, the formation of a Ru–Ge bond is anticipated, according to NMR spectra in solution. No crystals could be isolated. Upon addition of a solvent with donor properties such as acetonitrile, however, dissociation of the Ru fragment is observed, indicating the reversible nature of the first reaction step. In summary, we were able to show that the solvent as well as the steric demand of the ligands have a strong influence on the product formation. These results further underline the structural variability of metalated cluster species due to changes in the steric demand of the cluster core itself or through ligands bound to the metal atom. Finally, we showed the formation of [η^1^-Ge_9_(Si*^t^*Bu_2_H)_3_]RuCp(PPh_3_)_2_ (**2**), in which, for the first time, three covalently connected silyl ligands were bound to three neighboring Ge atoms, and that fluxional behavior and cluster atom rearrangements must be expected at all times.

## Figures and Tables

**Table 1:** Comparison between the geometrical parameters of **2**, **3b**, and cluster species known from the literature, concerning their three prism heights (h_1_, h_2_, h_3_), the ratio of the diagonals of the three pseudo square faces (d_1_/d_2_), the torsion angles of the pseudo square faces (α_1_, α_2_, α_3_), and the resulting cluster types.

Compound	Prism Heights/Å			d_1_/d_2_ ^(1)^			Torsion Angles/° ^(1)^			Cluster Type
	*h*_1_	*h*_2_	*h*_3_	*a*	*b*	*c*	α_1_	α_2_	α_3_	
[η^1^-Ge_9_(Si*^t^*Bu_2_H)_3_]RuCp(PPh_3_)_2_, **2**	3.700(2)	2.985(2)	3.068(1)	1.08	1.35	1.30	3.93(5)	29.48(5)	27.31(5)	*C*_4*v*_
[Hyp_3_Ge_9_][RuCp(PPh_3_)_2_(MeCN)], **3b**	3.2746(4)	3.2698(4)	3.6934(4)	1.13	1.13	1.08	26.78(2)	26.48(2)	14.80(2)	*D*_3*h*_
[(Si*^t^*Bu_2_H)_3_Ge_9_]^−^ [13]	3.4166(7)	3.3979(7)	3.3663(7)	1.05	1.08	1.07	22.78(2)	20.51(2)	23.93(2)	*D*_3*h*_
(η^3^-Ge_9_Hyp_3_)ZnCp* [28]	3.3509(6)	3.2478(7)	3.2994(6)	1.08	1.14	1.11	23.61(2)	26.09(2)	25.01(2)	*D*_3*h*_
Ge_9_Hyp_3_Et [17]	3.650(1)	3.0694(9)	3.0554(9)	1.06	1.26	1.26	3.39(3)	29.88(2)	32.17(3)	*C*_4*v*_
[Ge_9_Hyp_3_(COPh)] [15]	3.664(1)	3.124(1)	3.058(1)	1.05	1.24	1.28	3.02(4)	28.43(5)	32,33(4)	*C*_4*v*_
[Ge_9_Hyp_3_(CH_2_)_3_CH=CH_2_] [14]	3.6042(5)	3.1176(5)	3.1383(5)	1.02	1.24	1.22	4.98(2)	28.92(2)	30.23(2)	*C*_4*v*_
[(η^1^-Ge_9_Hyp_3_)Cr(CO)_5_]^−^ [18]	3.812(1)	3.090(1)	3.014(1)	1.14	1.23	1.29	5.21(2)	29.32(2)	32.08(2)	*C*_4*v*_

^(1)^ Ratios *a*, *b*, and *c*, as well as angles α_1_, α_2_, and α_3,_ are calculated with respect to the corresponding prism heights *h*_1_, *h*_2_, and *h*_3_, respectively.

## Data Availability

CCDC Deposition Numbers 2420650 (for **1**), 2420649 (for **2**), and 2420648 (for **3b**) contain the supplementary crystallographic data for this paper. These data can be obtained free of charge via www.ccdc.cam.ac.uk/data_request/cif accessed on 14 February 2025 (or from the CCDC, 12 Union Road, Cambridge CB2 1EZ, UK; Fax: +44-1223-336033; e-mail: deposit@ccdc.cam.ac.uk).

## Supplementary Materials

The following supporting information can be downloaded at: https://www.mdpi.com/article/10.3390/molecules30061247/s1, Figure S1: NMR spectrum of K[Hyp_3_Ge_9_]; Figure S2: NMR spectrum of K[(Si*^t^*Bu_2_H)_3_Ge_9_]; Figures S3–S7: NMR spectra of **1**; Figure S8: LIFDI/MS of **1**; Figures S9–S13: NMR spectra of **3a**; Figure S14: LIFDI/MS of **3a**; Figure S15: VT-NMR of **3a**; Figures S16 and S17: NMR spectra of the reaction of **3a** to **3b**; Figures S18–S20: Unit cells of **1**, **2**, and **3b**; Tables S1–S6: Bond lengths and bond angles of **1**, **2**, and **3b**; Figure S21: Crystallization setup **3b**.
