# Supplementary material for: Ruthenium Decorated Tris-Silylated Germanium Zintl Clusters Featuring an Unexpected Ligand Arrangement
Source: Molecules. 2025 Mar 11;30(6):1247. doi: 10.3390/molecules30061247 (PMC11946829; doi:10.3390/molecules30061247)
Supplement: Supplementary file 1 [file molecules-30-01247-s001.zip › molecules-3504370-supplementary.pdf]

Supporting Information

Content

Characterization 2

$K[Hyp_3Ge_9]$ 2

$K[(^tBu_2HSi)_3Ge_9]$ 3

$[\eta^5-Ge_9Hyp_3]RuCp^*$ (1) 4

$[Hyp_3Ge_9][RuCp(PPh_3)_2]$ (3a) or $[Hyp_3Ge_9][RuCp(PPh_3)_2(MeCN)]$ (3b) 8

Crystallographic details 13

$[\eta^5-Ge_9Hyp_3]RuCp^*$ (1) 14

$[\eta^1-Ge_9(Si^tBu_2H)_3]RuCp(PPh_3)_2$ (2) 18

$[Hyp_3Ge_9][RuCp(PPh_3)_2(MeCN)]$ (3b) 22

Characterization

K[Hyp$_3$Ge$_9$]

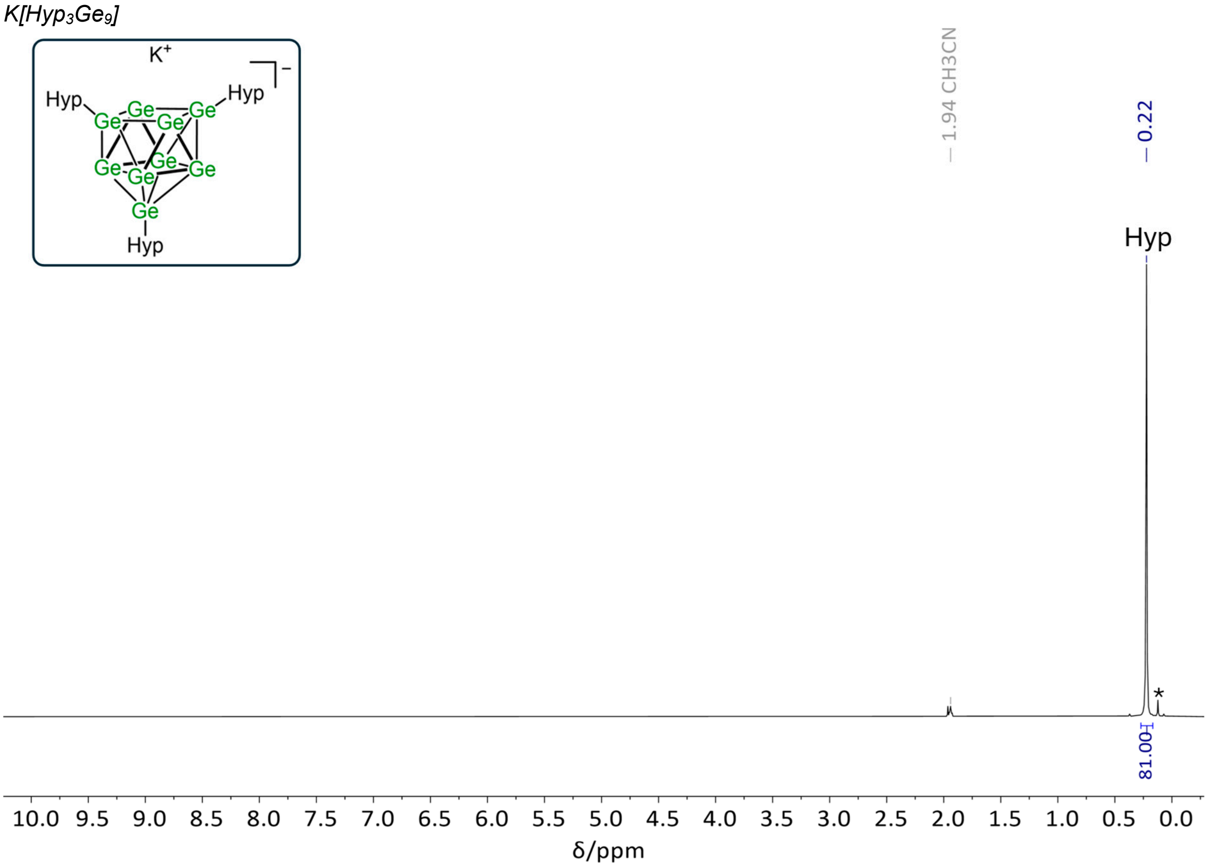

Figure S1. ^{1}H NMR spectrum (400 MHz) of K[Hyp$_3$Ge$_9$] recorded in acetonitrile-*d*$_3$ at r.t. The signal marked with * could not be assigned.

K[(^{t}Bu$_2$HSi)$_3$Ge$_9$]

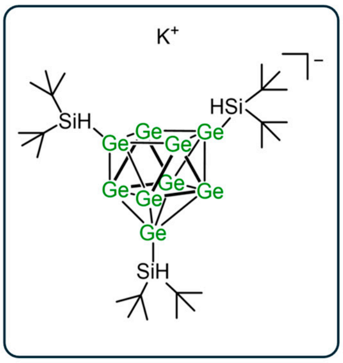

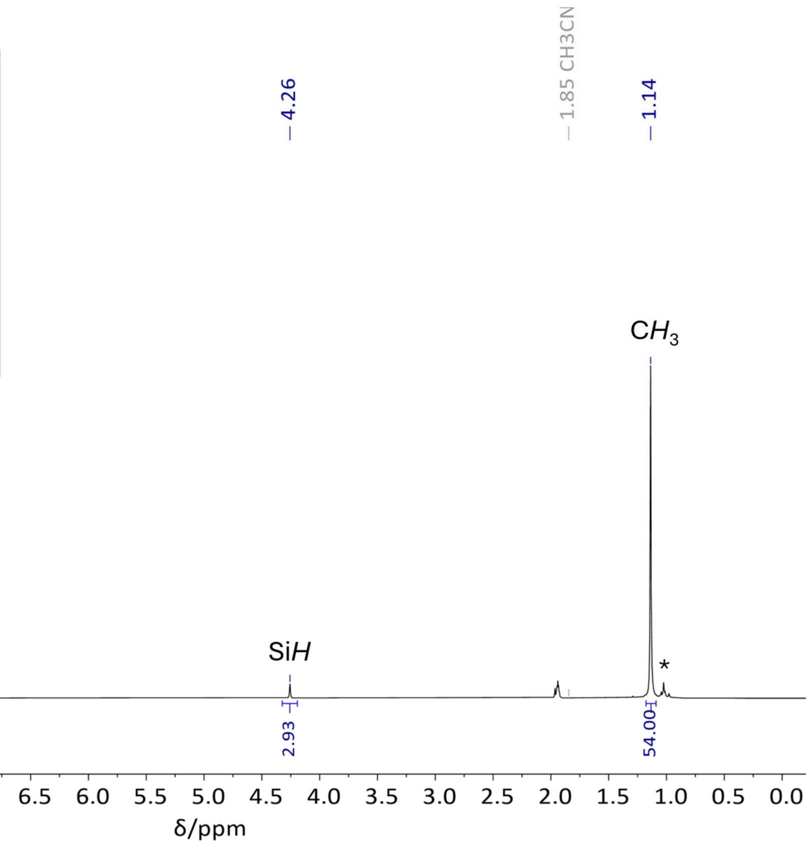

Figure S2. ^{1}H NMR spectrum (400 MHz) of K[(^{t}Bu$_2$HSi)$_3$Ge$_9$] recorded in acetonitrile-d_3 at r.t. The signal marked with * could not be assigned.

[η⁵-Ge₉Hyp₃]RuCp (1)*

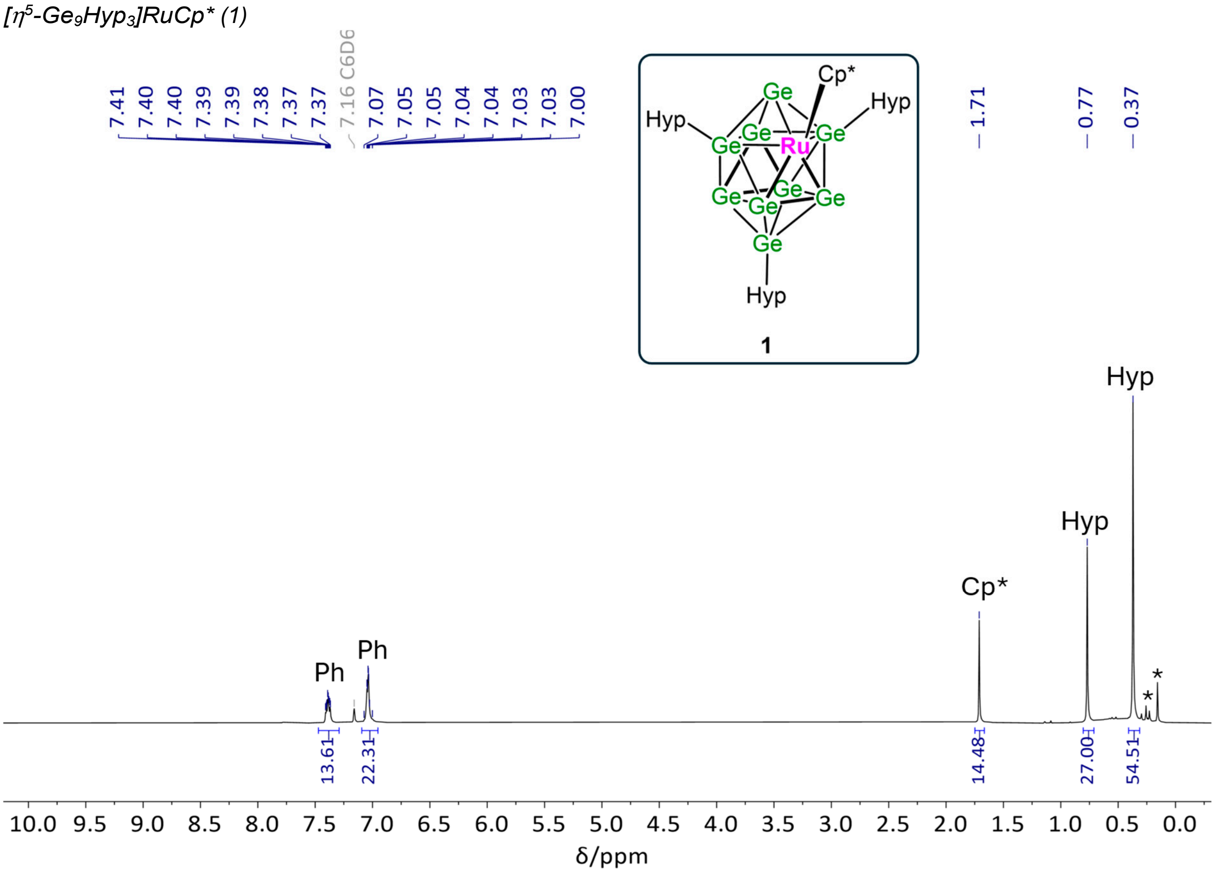

Figure S3. ^{1}H NMR spectrum (400 MHz) of **1** recorded in C_6D_6 at r.t. The signals marked with * could not be assigned.

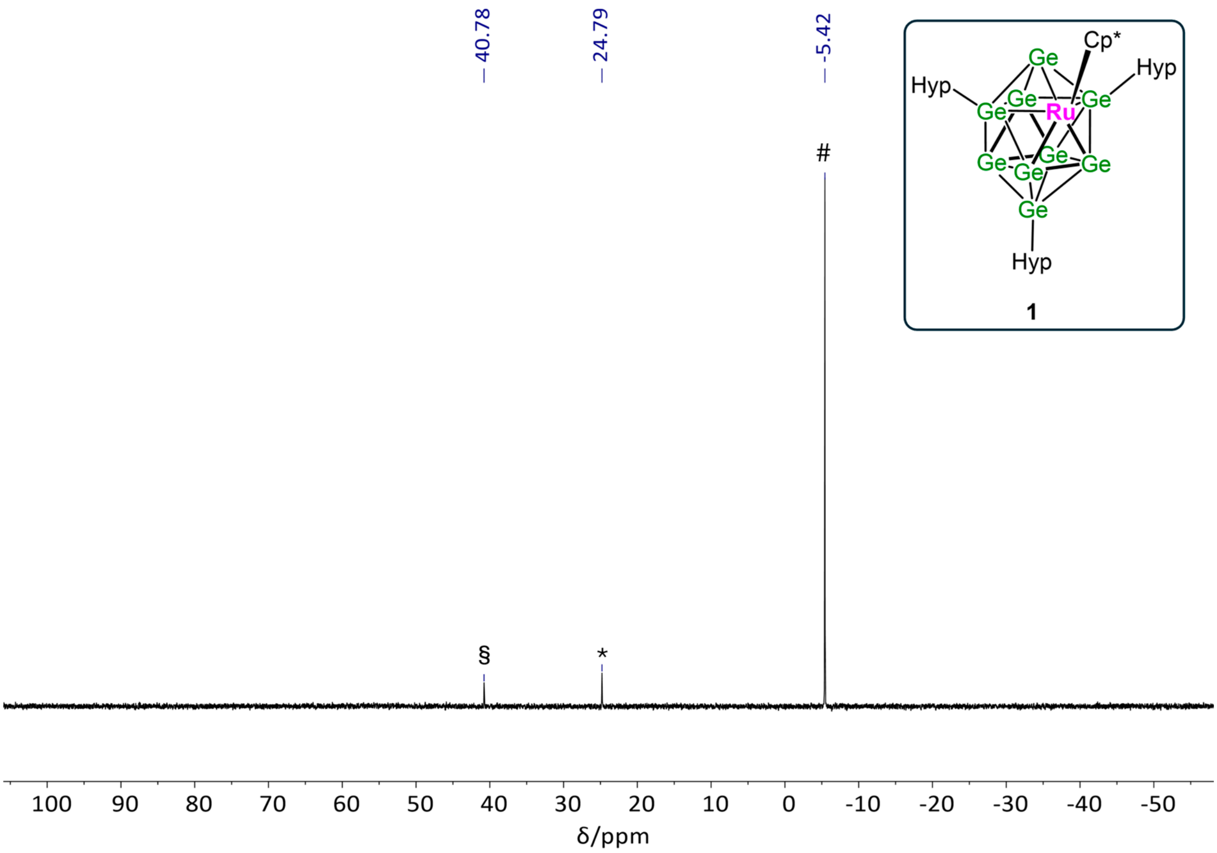

Figure S4. $^{31}P\{^{1}H\}$ NMR spectrum (162 MHz) of **1** recorded in C_6D_6 at r.t. The signal marked with # comes from unattached PPh_3, the signal marked with § comes from the reagent $RuCp^*(PPh_3)_2Cl$ and the signals marked with * could not be assigned.

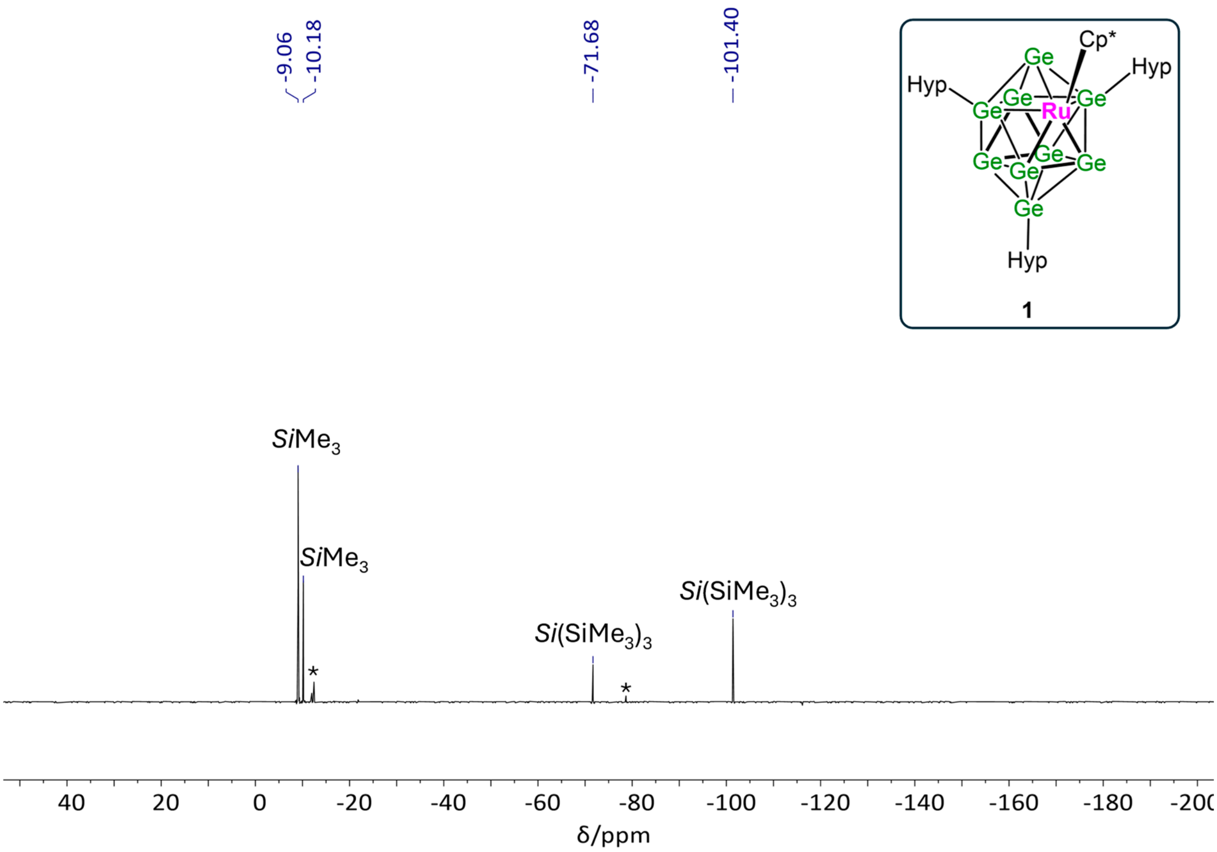

Figure S5. $^{29}Si\{^{1}H\}$ NMR spectrum (162 MHz) of **1** recorded in C_6D_6 at r.t. The signals marked with * could not be assigned.

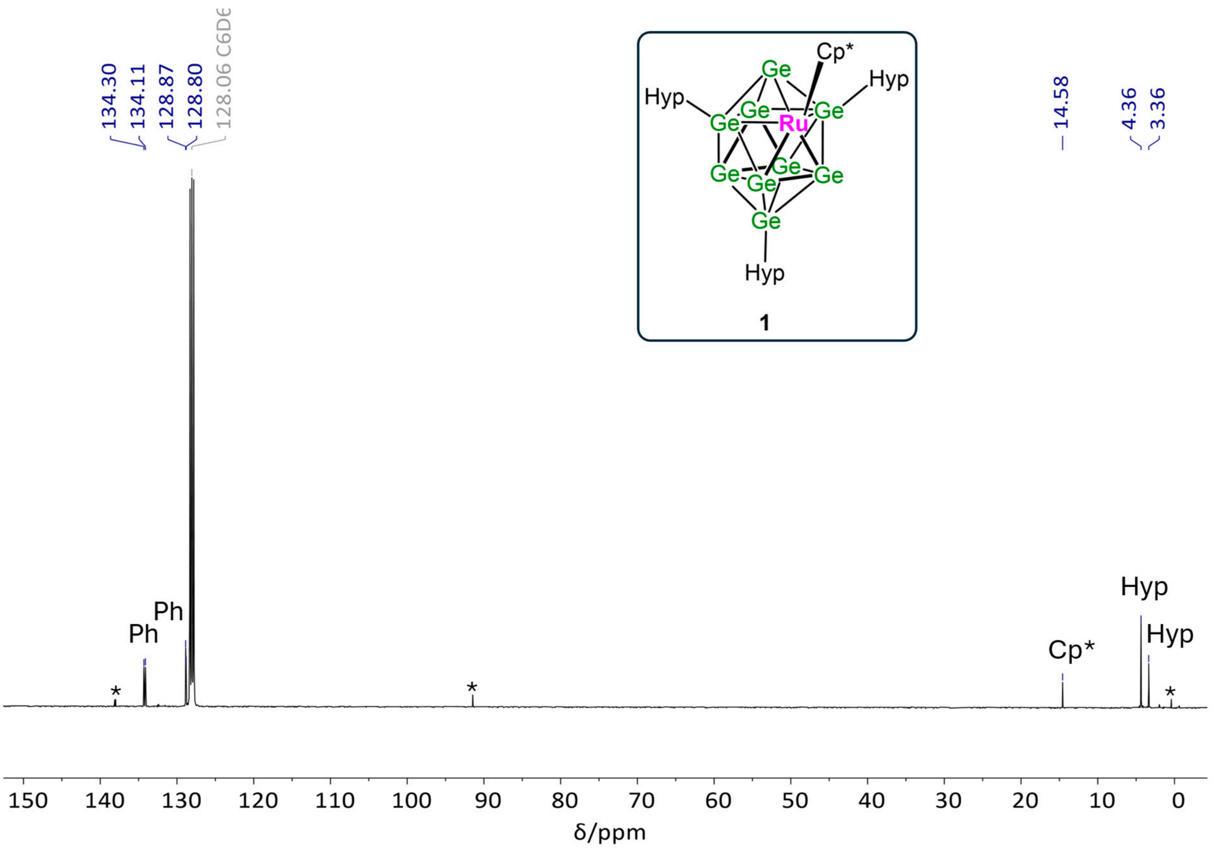

Figure S6. $^{13}C\{^{1}H\}$ NMR spectrum (100 MHz) of **1** recorded in C_6D_6 at r.t. The signals marked with * could not be assigned.

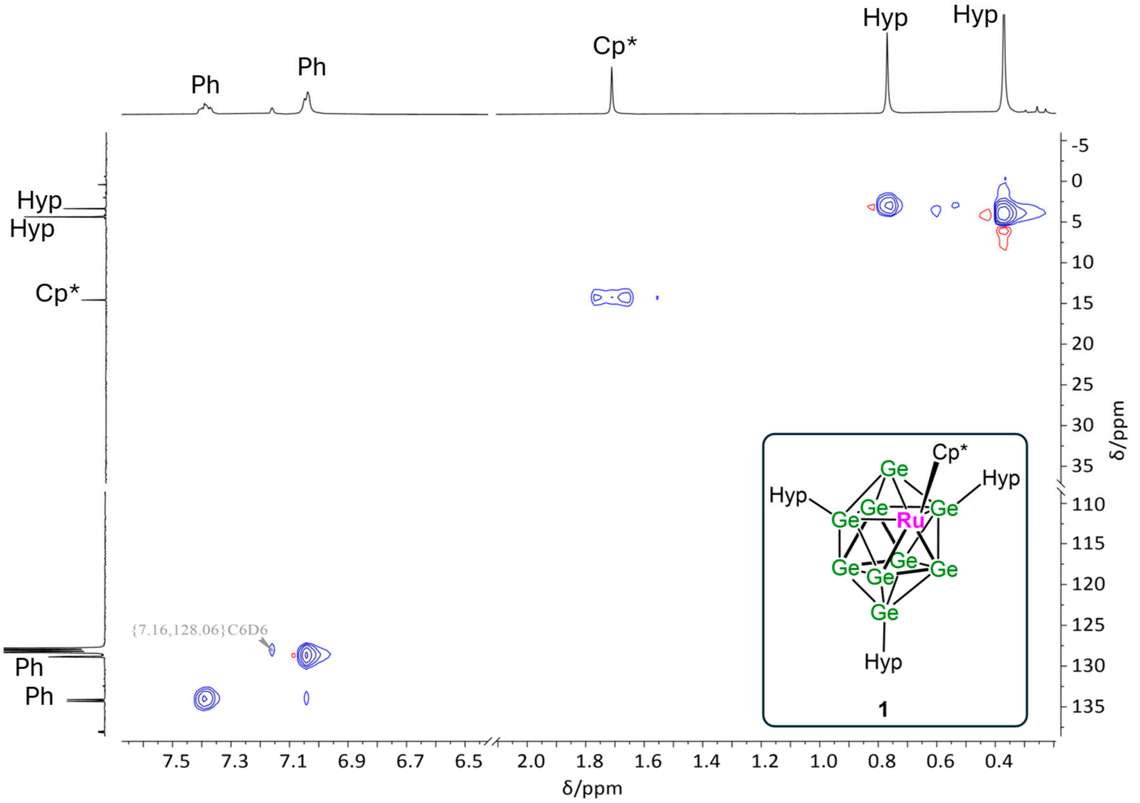

Figure S7. $^{1}H^{13}C$ HSQC NMR spectrum (400 MHz, 100 MHz) of **1** recorded in C_6D_6 at r.t.

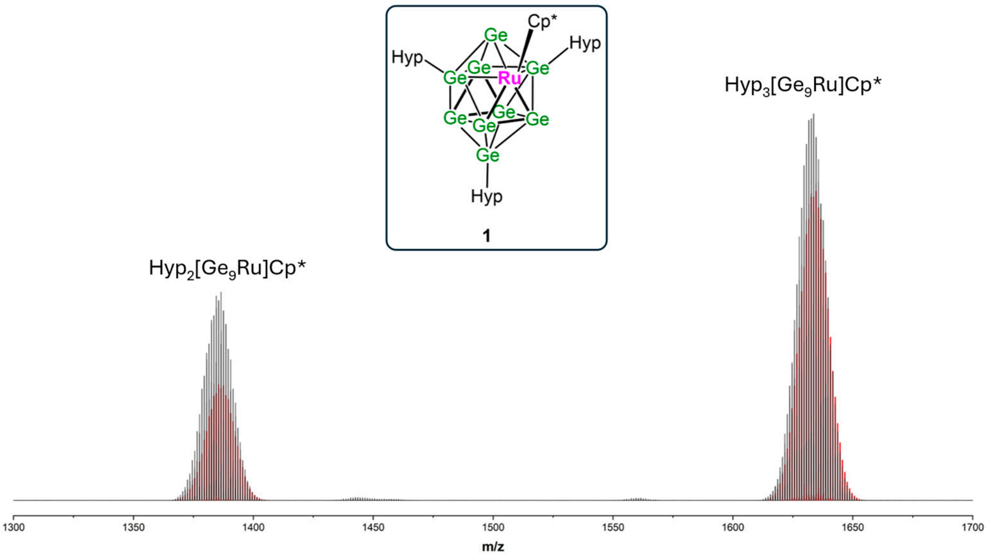

Figure S8. LIFDI mass spectrum of **1** in black and simulated isotope pattern in red.

[Hyp_3Ge_9][$RuCp(PPh_3)_2$] (3a) or [Hyp_3Ge_9][$RuCp(PPh_3)_2(MeCN)$] (3b)

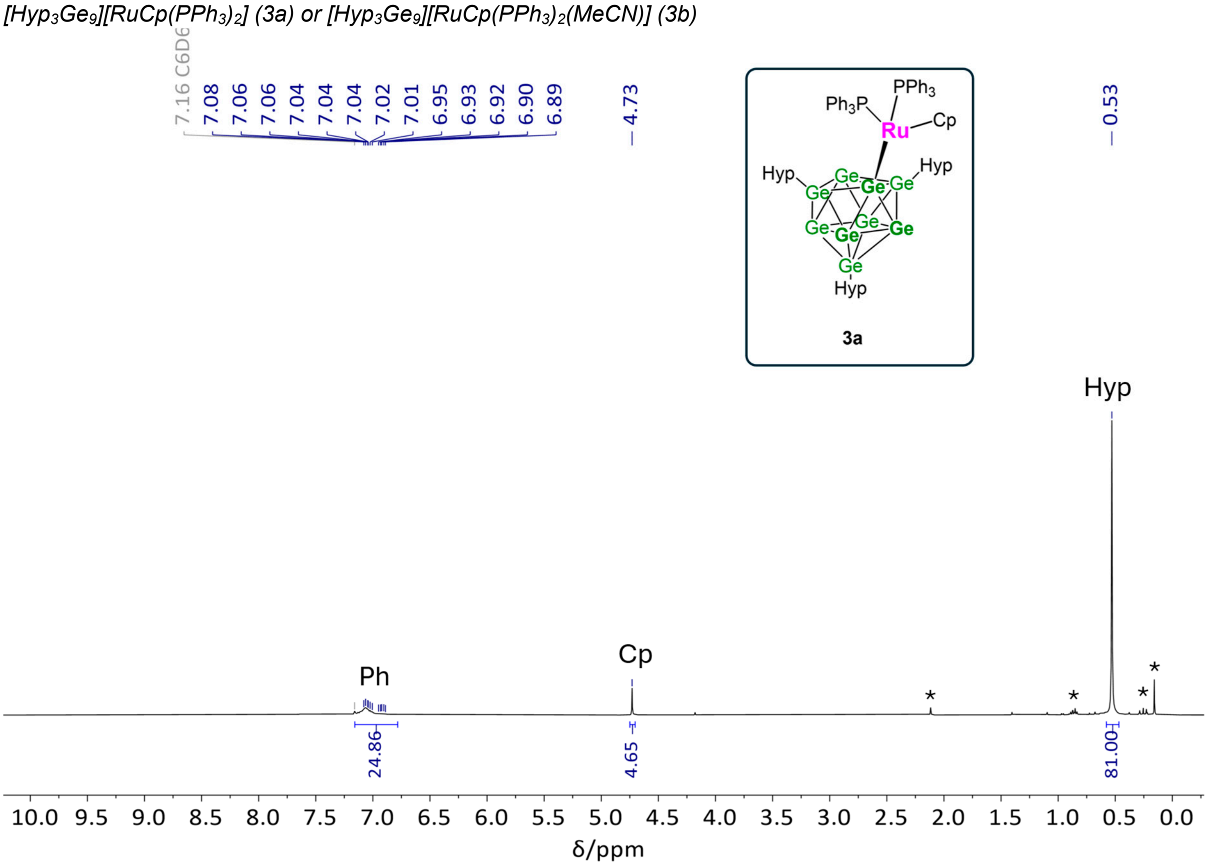

Figure S9. ^{1}H NMR spectrum (400 MHz) of **3a** recorded in C_6D_6 at r.t. The signals marked with * could not be assigned.

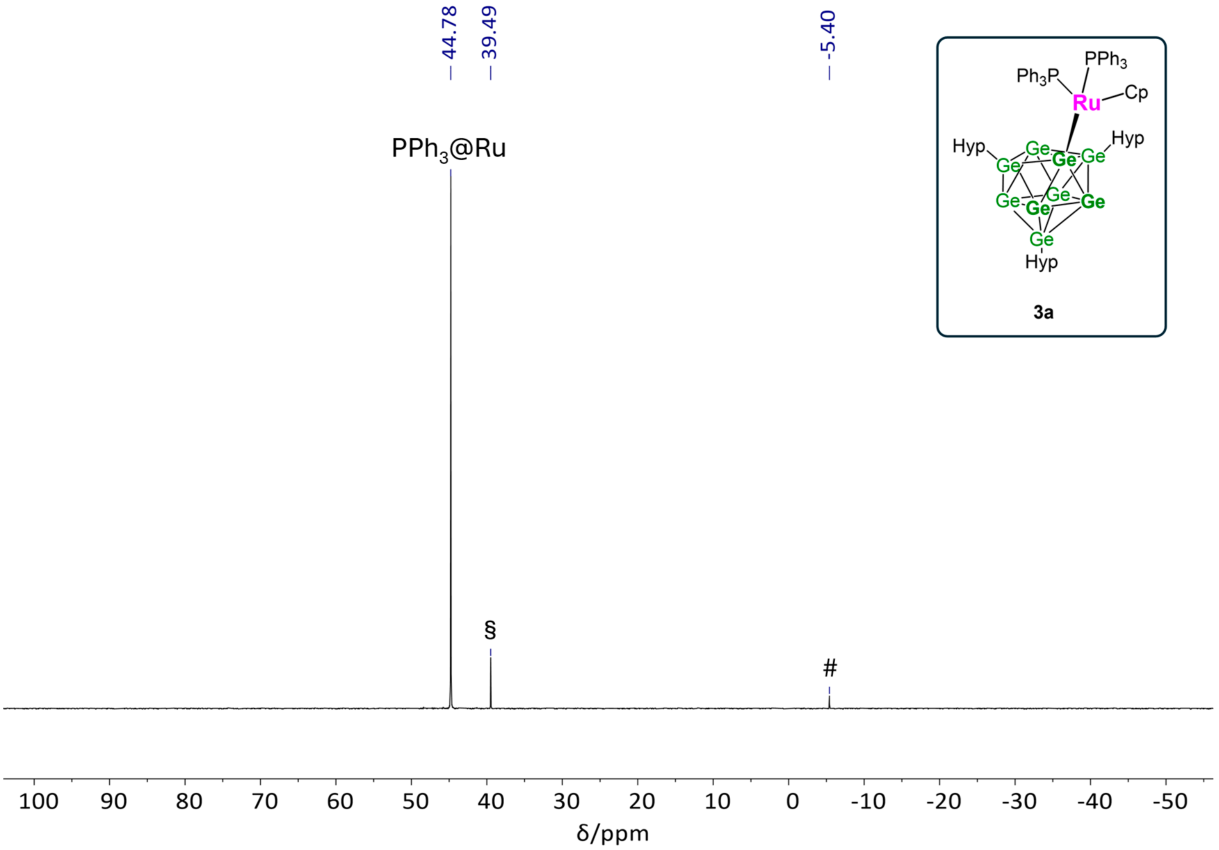

Figure S10. $^{31}P\{^{1}H\}$ NMR spectrum (162 MHz) of **3a** recorded in C_6D_6 at r.t. The signal marked with # comes from unattached PPh_3 and the signal marked with § comes from the reagent $RuCp(PPh_3)_2Cl$.

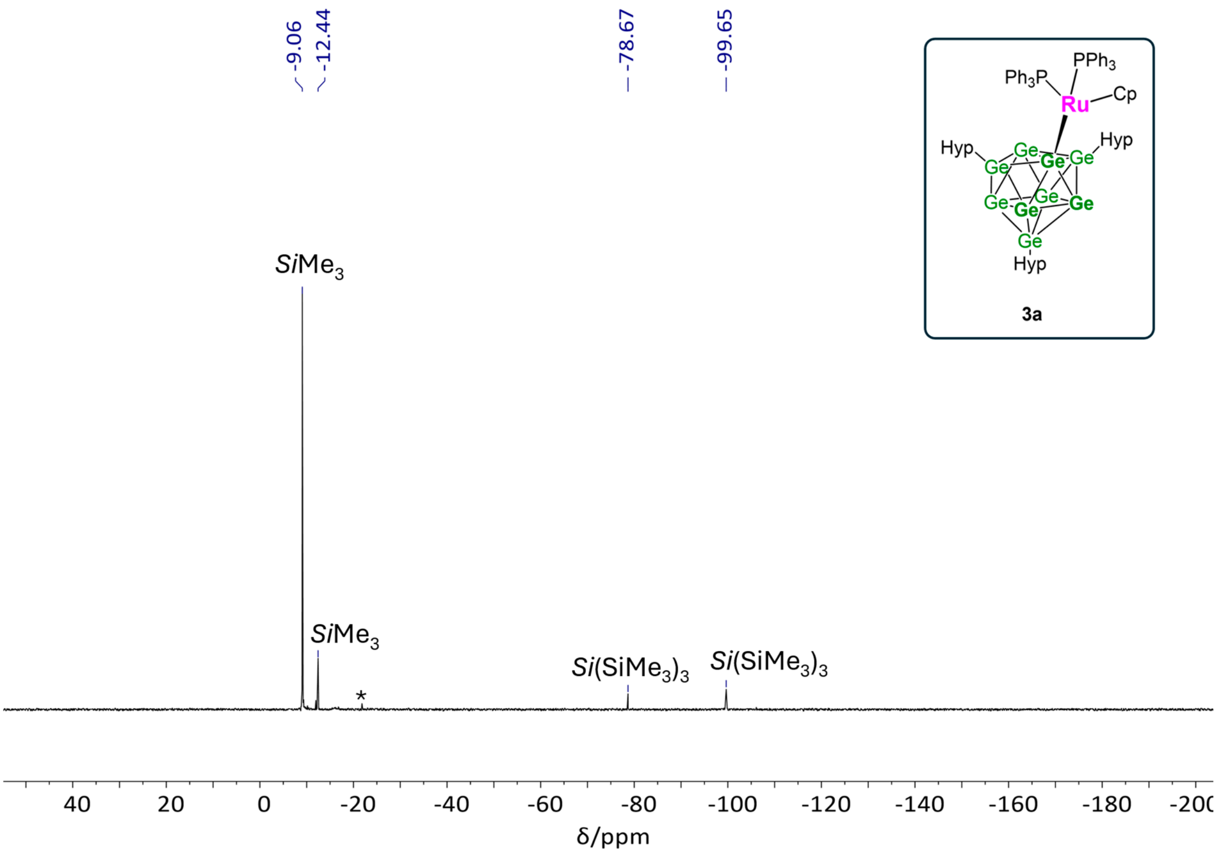

Figure S11. $^{29}Si\{^1H\}$ NMR spectrum (162 MHz) of **3a** recorded in C_6D_6 at r.t. The signal marked with * could not be assigned.

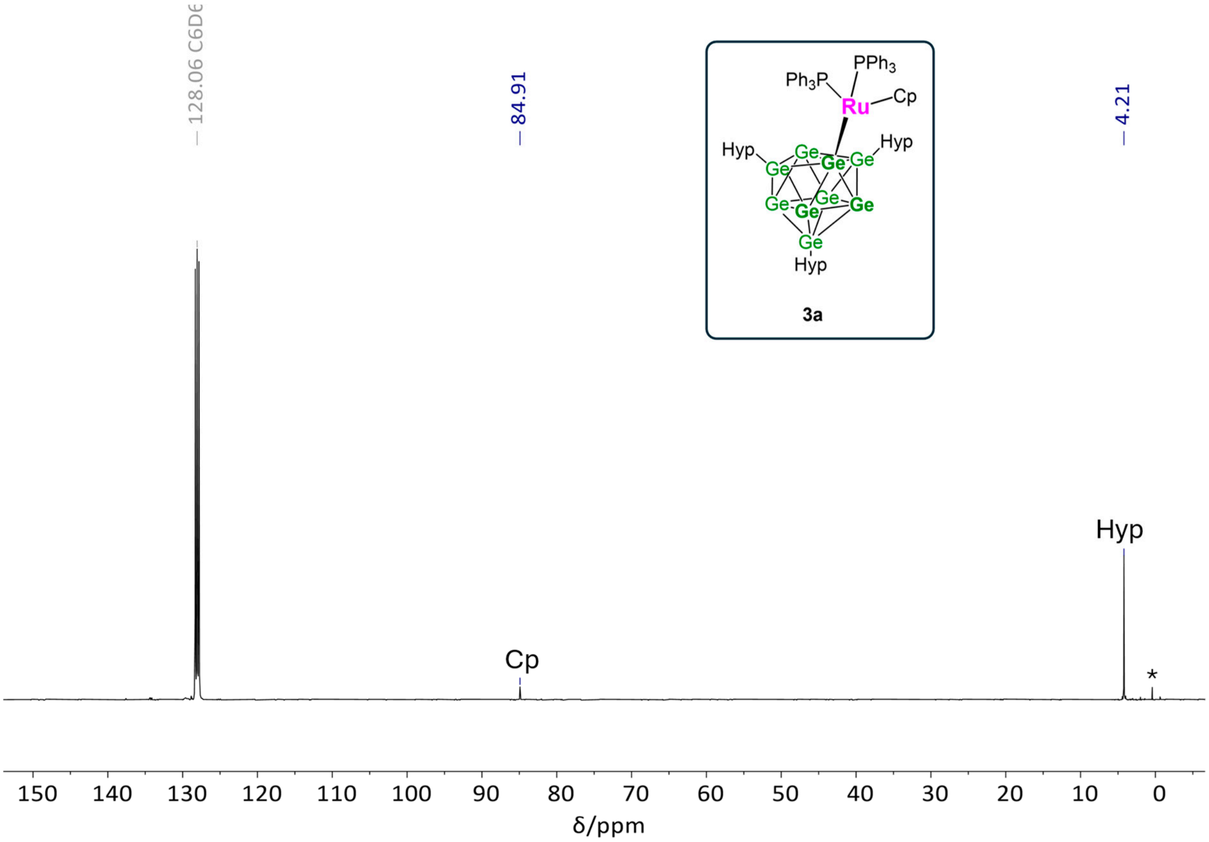

Figure S12. $^{13}C\{^1H\}$ NMR spectrum (100 MHz) of **3a** recorded in C_6D_6 at r.t. The signal marked with * could not be assigned.

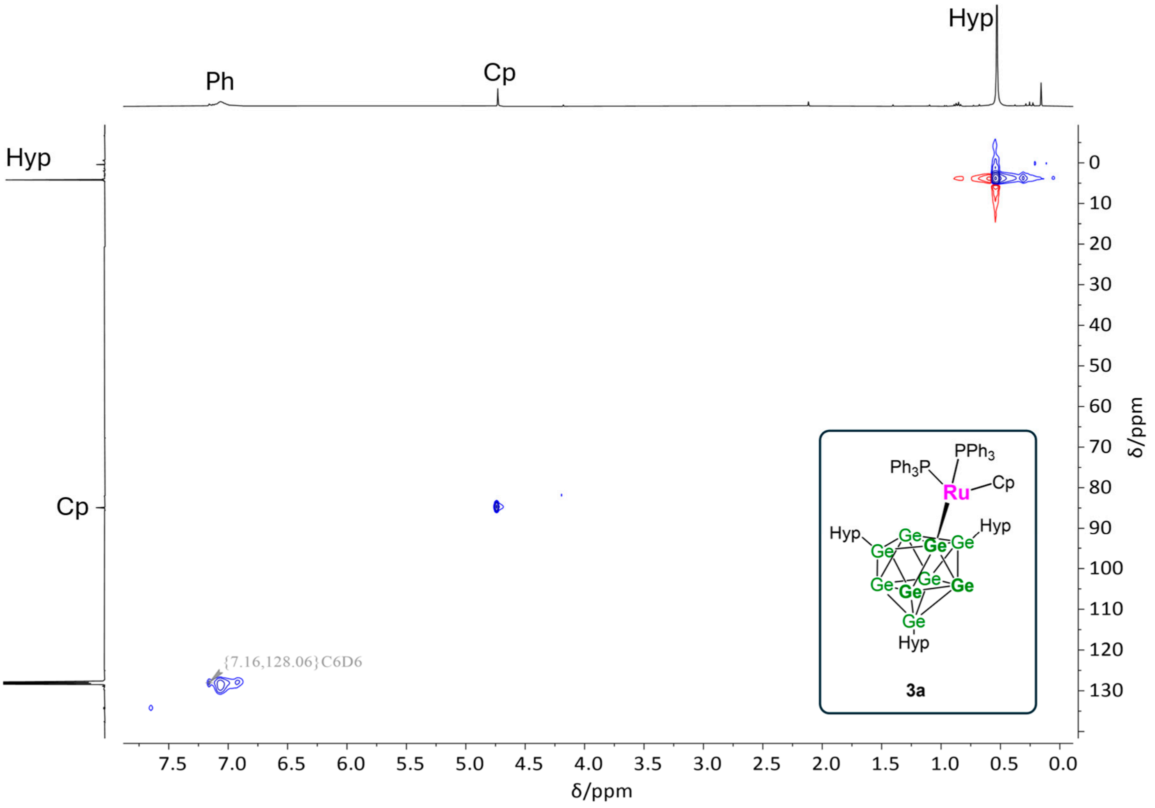

Figure S13. $^{1}H^{13}C$ HSQC NMR spectrum (400 MHz, 100 MHz) of **3a** recorded in C_6D_6 at r.t.

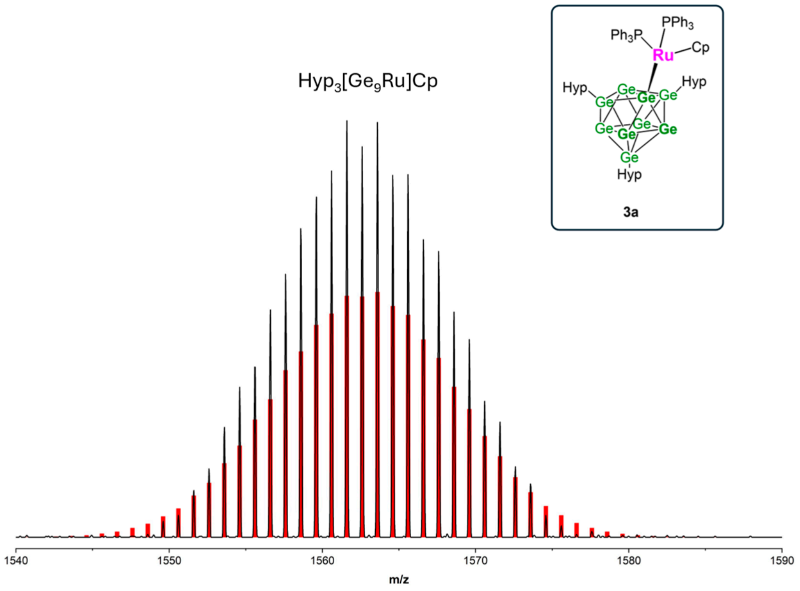

Figure S14. LIFDI mass spectrum of **3a** in black and simulated isotope pattern in red.

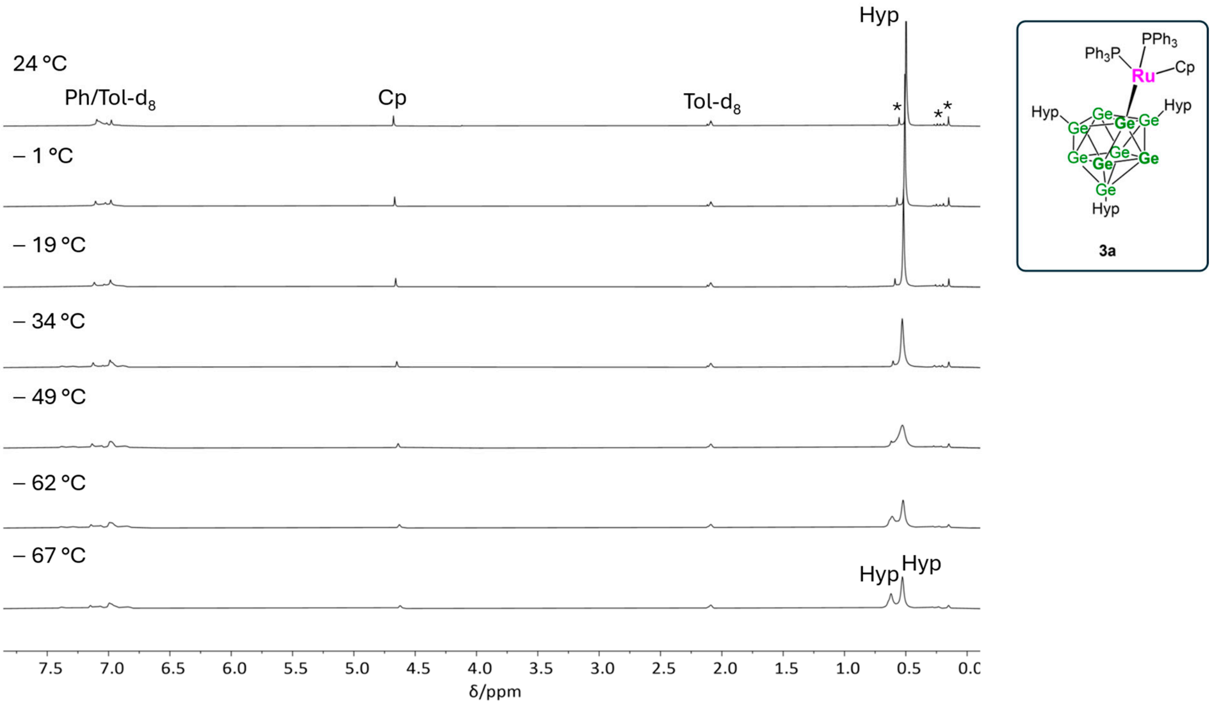

Figure S15. Variable temperature ^{1}H NMR spectra (400 MHz) of **3a** recorded in toluene-d_8 from 24 °C to – 67 °C. The signals marked with * could not be assigned.

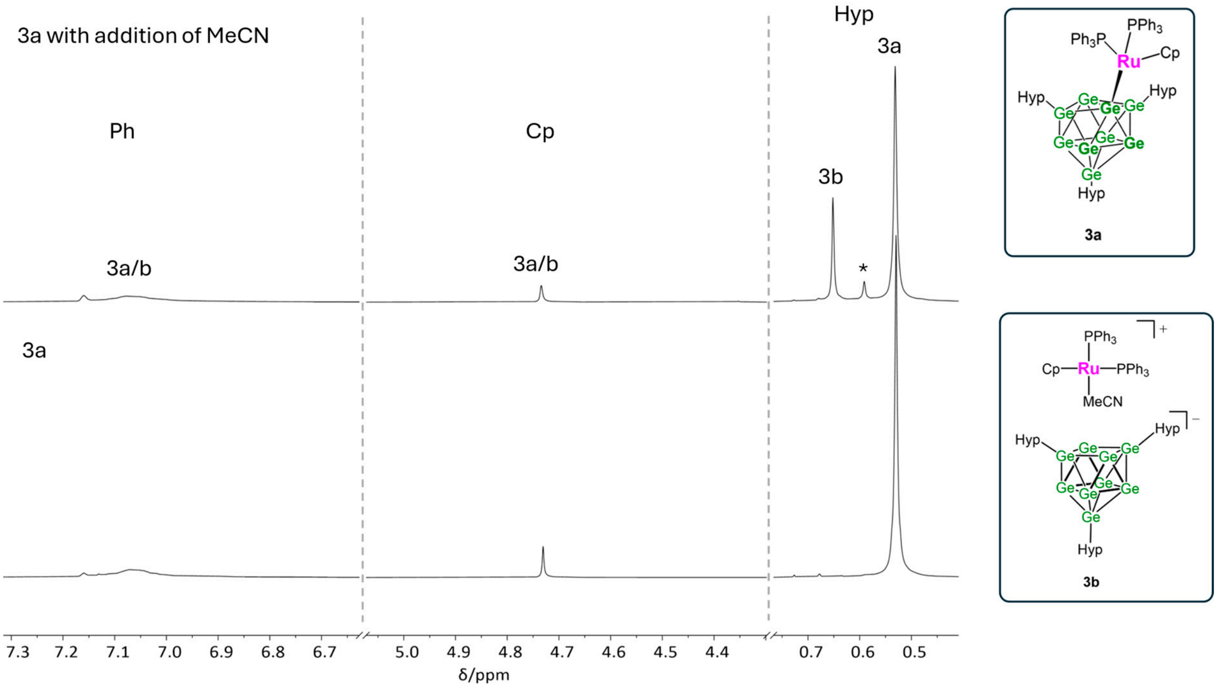

Figure S16. ^{1}H NMR spectra (400 MHz) of **3a** and the reaction between **3a** and acetonitrile to yield **3b** recorded in C_6D_6 at r.t. The signal marked with * could not be assigned.

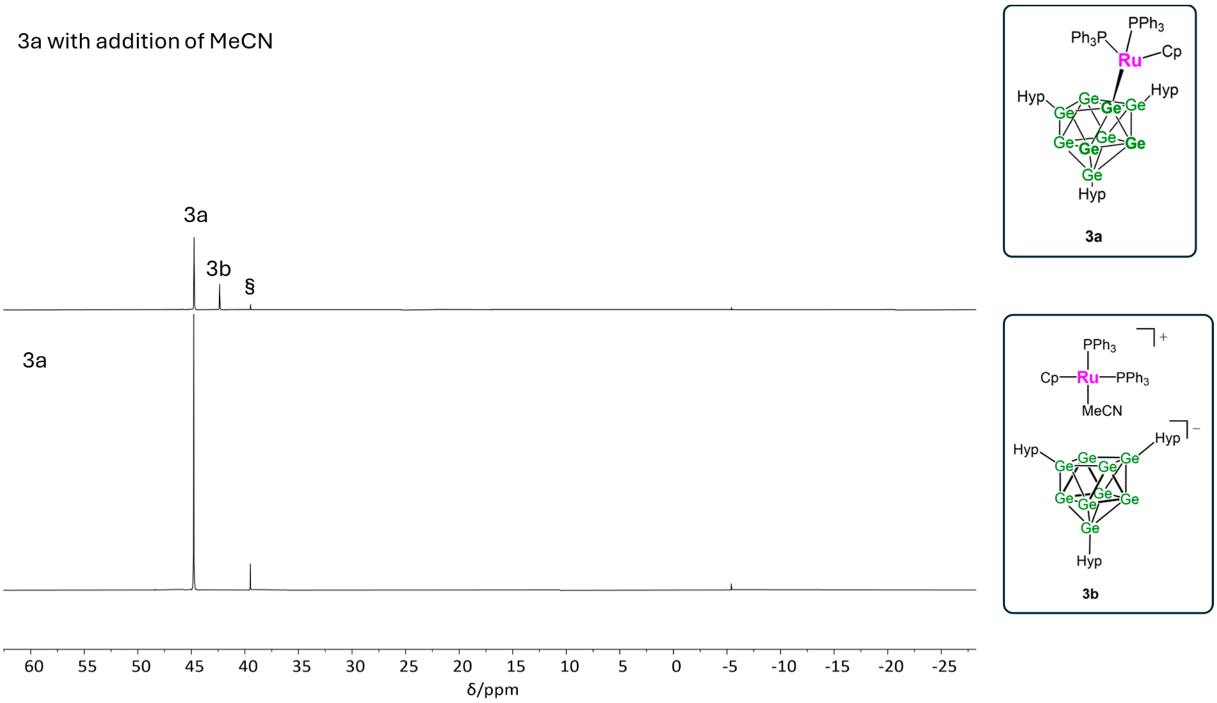

Figure S17. $^{31}P\{^1H\}$ NMR spectra (162 MHz) of **3a** and the reaction between **3a** and acetonitrile to yield **3b** recorded in C_6D_6 at r.t. The signal marked with § comes from the reagent $RuCp(PPh_3)_2Cl$.

Crystallographic details

Compound	1	2	3b
formula	$C_{37}H_{97}Ge_9RuSi_{12}$	$C_{65}H_{92}Ge_9P_2RuSi_3$	$C_{68}H_{116}Ge_9P_2RuSi_{12}$
fw/g·mol^{-1}	1632.59	1921.20	2210.16
space group	C c	P –1	P –1
a/Å	15.8985(4)	15.9385(6)	13.4786(2)
b/Å	26.4490(6)	16.4463(7)	17.4875(3)
c/Å	18.2700(6)	17.7098(7)	24.1636(5)
a/deg	90	73.695(3)	81.0890(10)
b/deg	112.075(2)	67.117(3)	85.204(2)
g/deg	90	89.466(3)	67.9950(10)
V/Å^3	7119.3(3)	4078.6(3)	5214.85(17)
Z	4	2	2
T/K	150(2)	150(2)	150(2)
l/Å	0.71073	0.71073	0.71073
ρ_{calcd}/g·cm^{-3}	1.523	1.564	1.408
µ/mm^{-1}	4.173	3.569	2.899
collected reflections	42380	61077	72063
indep. reflections	13305	18727	23779
reflections with I > 2σ(I)	9953	8849	19297
R_{int}/R_d	0.0535	0.1021	0.0215
parameters/ restraints	603/20	856/0	941/0
R_1 [I>2s(I) / all data]	0.0503/0.0822	0.0741/0.1820	0.0431/0.0571
wR_2 [I>2s(I) / all data]	0.0983/0.1087	0.1389/0.1716	0.1088/0.1172
goodness of fit	1.012	0.975	1.014
max./min. diff. el. densitiy/e·Å^3	0.793/–0.474	0.873/–0.741	0.789/–1.083
CCDC	2420605	2420649	2420648

[η^5-Ge_9Hyp_3]RuCp (1)*

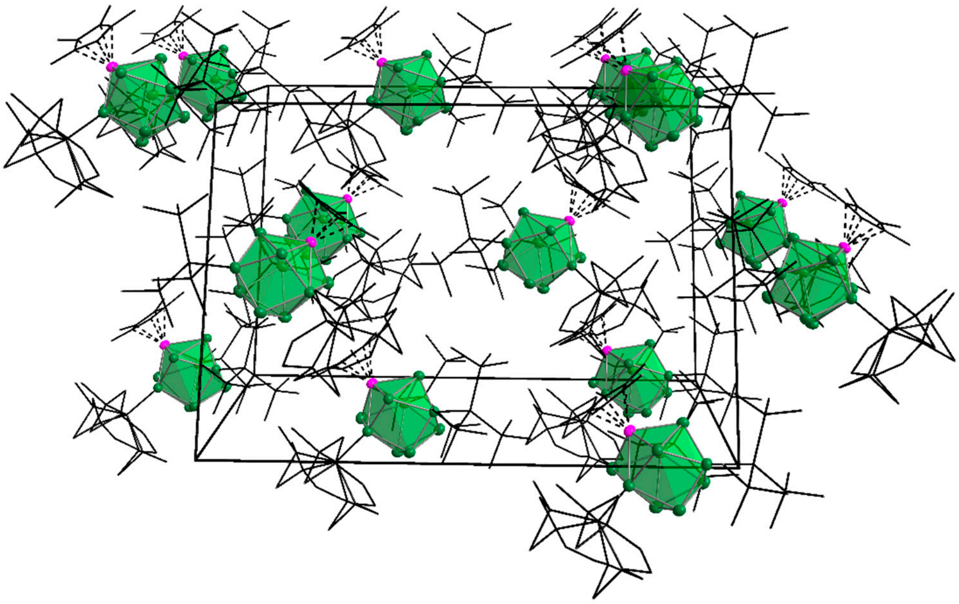

Figure S18. Unit cell of **1**. Cluster cores [Ge_9Ru] are shown as green polyhedra. All ligands (hypersilyl, cyclopentadienyl) are simplified for clarity reasons. Germanium and ruthenium atoms are depicted in green and pink color.

Table S1. Bond lengths in [Å] in **1**. The numbers of the various residues are shown here using underscores. Residue 1 is the cluster core [Ge_9Ru], residues 2, 3 and 4 are the silyl ligands and residue 5 is the pentamethylcyclopentadienyl ligand.

Atom 1	Atom 2	Bond length	Atom 1	Atom 2	Bond length
Ru1_1	C4_5	2.212(14)	Si4A_2	C7A_2	1.95(2)
Ru1_1	C1_5	2.217(11)	Si4B_2	C4_2	1.74(2)
Ru1_1	C3_5	2.227(11)	Si4B_2	C8B_2	1.85(5)
Ru1_1	C2_5	2.235(12)	Si4B_2	C3_2	2.08(2)
Ru1_1	C5_5	2.251(14)	Si2B_2	C2_2	1.57(3)
Ru1_1	Ge4_1	2.5752(14)	Si2B_2	C9B_2	1.88(4)
Ru1_1	Ge8_1	2.5793(13)	Si2B_2	C1_2	2.11(2)
Ru1_1	Ge7_1	2.5972(13)	Si3B_2	C6_2	1.72(2)
Ru1_1	Ge3_1	2.6705(14)	Si3B_2	C7B_2	1.88(5)
Ru1_1	Ge1_1	2.6765(13)	Si3B_2	C5_2	1.97(2)
Ge1_1	Si1_3	2.435(3)	Si1_3	Si2_3	2.364(4)
Ge1_1	Ge4_1	2.5243(16)	Si1_3	Si4_3	2.365(5)
Ge1_1	Ge8_1	2.5579(15)	Si1_3	Si3_3	2.372(5)
Ge1_1	Ge5_1	2.6082(15)	Si2_3	C1_3	1.805(16)
Ge1_1	Ge2_1	2.7089(18)	Si2_3	C3_3	1.850(15)
Ge2_1	Ge5_1	2.6163(17)	Si2_3	C2_3	1.880(17)
Ge2_1	Ge6_1	2.6391(16)	Si3_3	C4_3	1.83(2)
Ge2_1	Ge3_1	2.7018(15)	Si3_3	C6_3	1.869(19)
Ge2_1	Ge4_1	2.7852(17)	Si3_3	C5_3	1.891(13)
Ge3_1	Si1_2	2.443(3)	Si4_3	C9_3	1.818(18)
Ge3_1	Ge4_1	2.5411(16)	Si4_3	C7_3	1.861(14)
Ge3_1	Ge7_1	2.5509(15)	Si4_3	C8_3	1.88(2)
Ge3_1	Ge6_1	2.6111(15)	Si1_4	Si3_4	2.346(5)
Ge5_1	Ge9_1	2.5001(15)	Si1_4	Si2_4	2.350(4)
Ge5_1	Ge6_1	2.7962(16)	Si1_4	Si4_4	2.355(4)
Ge6_1	Ge9_1	2.5110(16)	Si2_4	C1_4	1.860(13)
Ge7_1	Ge9_1	2.5386(14)	Si2_4	C2_4	1.868(19)
Ge7_1	Ge8_1	2.8171(15)	Si2_4	C3_4	1.893(16)
Ge8_1	Ge9_1	2.5421(16)	Si3_4	C4_4	1.864(13)
Ge9_1	Si1_4	2.387(3)	Si3_4	C5_4	1.885(14)
Si1_2	Si3B_2	2.214(13)	Si3_4	C6_4	1.892(14)
Si1_2	Si2A_2	2.330(5)	Si4_4	C8_4	1.823(14)
Si1_2	Si4B_2	2.362(12)	Si4_4	C9_4	1.853(17)
Si1_2	Si2B_2	2.395(13)	Si4_4	C7_4	1.861(15)
Si1_2	Si3A_2	2.405(6)	C1_5	C2_5	1.38(2)
Si1_2	Si4A_2	2.419(7)	C1_5	C6_5	1.47(2)
Si2A_2	C1_2	1.809(15)	C1_5	C3_5	1.52(2)
Si2A_2	C2_2	1.84(3)	C2_5	C5_5	1.35(2)
Si2A_2	C9A_2	1.89(2)	C2_5	C7_5	1.50(2)
Si3A_2	C6_2	1.787(18)	C3_5	C4_5	1.41(3)
Si3A_2	C5_2	1.841(17)	C3_5	C10_5	1.46(2)
Si3A_2	C8A_2	1.87(3)	C4_5	C5_5	1.34(2)
Si4A_2	C4_2	1.81(2)	C4_5	C9_5	1.54(3)
Si4A_2	C3_2	1.906(17)	C5_5	C8_5	1.55(2)

Table S2. Bond angles in [°] in **1**. The numbers of the various residues are shown here using underscores. Residue 1 is the cluster core [Ge_9Ru], residues 2, 3 and 4 are the silyl ligands and residue 5 is the pentamethylcyclopentadienyl ligand.

Atom 1	Atom 2	Atom 3	Bond angle	Atom 1	Atom 2	Atom 3	Bond angle
C4_5	Ru1_1	C1_5	63.0(5)	Si3B_2	Si1_2	Si2B_2	115.0(6)
C4_5	Ru1_1	C3_5	37.0(7)	Si4B_2	Si1_2	Si2B_2	103.7(5)
C1_5	Ru1_1	C3_5	39.9(6)	Si2A_2	Si1_2	Si3A_2	110.2(2)
C4_5	Ru1_1	C2_5	58.6(5)	Si2A_2	Si1_2	Si4A_2	104.5(3)
C1_5	Ru1_1	C2_5	36.1(5)	Si3A_2	Si1_2	Si4A_2	102.5(3)
C3_5	Ru1_1	C2_5	61.0(5)	Si3B_2	Si1_2	Ge3_1	110.7(4)
C4_5	Ru1_1	C5_5	34.9(6)	Si2A_2	Si1_2	Ge3_1	114.7(2)
C1_5	Ru1_1	C5_5	61.4(5)	Si4B_2	Si1_2	Ge3_1	104.9(3)
C3_5	Ru1_1	C5_5	60.8(5)	Si2B_2	Si1_2	Ge3_1	108.5(3)
C2_5	Ru1_1	C5_5	35.0(5)	Si3A_2	Si1_2	Ge3_1	113.88(19)
C4_5	Ru1_1	Ge4_1	96.6(4)	Si4A_2	Si1_2	Ge3_1	109.92(18)
C1_5	Ru1_1	Ge4_1	122.5(5)	C1_2	Si2A_2	C2_2	111.6(11)
C3_5	Ru1_1	Ge4_1	90.6(4)	C1_2	Si2A_2	C9A_2	110.7(9)
C2_5	Ru1_1	Ge4_1	151.3(3)	C2_2	Si2A_2	C9A_2	94.2(14)
C5_5	Ru1_1	Ge4_1	128.6(4)	C1_2	Si2A_2	Si1_2	112.6(6)
C4_5	Ru1_1	Ge8_1	114.6(6)	C2_2	Si2A_2	Si1_2	117.9(9)
C1_5	Ru1_1	Ge8_1	123.1(5)	C9A_2	Si2A_2	Si1_2	108.1(8)
C3_5	Ru1_1	Ge8_1	146.8(4)	C6_2	Si3A_2	C5_2	112.4(10)
C2_5	Ru1_1	Ge8_1	90.8(3)	C6_2	Si3A_2	C8A_2	104.8(13)
C5_5	Ru1_1	Ge8_1	86.1(4)	C5_2	Si3A_2	C8A_2	105.4(10)
Ge4_1	Ru1_1	Ge8_1	114.35(5)	C6_2	Si3A_2	Si1_2	112.0(7)
C4_5	Ru1_1	Ge7_1	146.2(4)	C5_2	Si3A_2	Si1_2	110.2(6)
C1_5	Ru1_1	Ge7_1	87.8(4)	C8A_2	Si3A_2	Si1_2	111.8(9)
C3_5	Ru1_1	Ge7_1	124.9(6)	C4_2	Si4A_2	C3_2	102.8(9)
C2_5	Ru1_1	Ge7_1	87.9(3)	C4_2	Si4A_2	C7A_2	105.0(11)
C5_5	Ru1_1	Ge7_1	117.1(4)	C3_2	Si4A_2	C7A_2	113.5(11)
Ge4_1	Ru1_1	Ge7_1	114.33(5)	C4_2	Si4A_2	Si1_2	118.1(7)
Ge8_1	Ru1_1	Ge7_1	65.94(4)	C3_2	Si4A_2	Si1_2	106.9(6)
C4_5	Ru1_1	Ge3_1	143.6(6)	C7A_2	Si4A_2	Si1_2	110.5(9)
C1_5	Ru1_1	Ge3_1	106.7(4)	C4_2	Si4B_2	C8B_2	102.1(17)
C3_5	Ru1_1	Ge3_1	111.8(5)	C4_2	Si4B_2	C3_2	98.5(10)
C2_5	Ru1_1	Ge3_1	133.9(4)	C8B_2	Si4B_2	C3_2	121.0(17)
C5_5	Ru1_1	Ge3_1	167.9(4)	C4_2	Si4B_2	Si1_2	124.4(9)
Ge4_1	Ru1_1	Ge3_1	57.92(4)	C8B_2	Si4B_2	Si1_2	108.8(18)
Ge8_1	Ru1_1	Ge3_1	100.31(4)	C3_2	Si4B_2	Si1_2	103.2(7)
Ge7_1	Ru1_1	Ge3_1	57.91(4)	C2_2	Si2B_2	C9B_2	101(2)
C4_5	Ru1_1	Ge1_1	108.7(4)	C2_2	Si2B_2	C1_2	109.3(13)
C1_5	Ru1_1	Ge1_1	171.7(4)	C9B_2	Si2B_2	C1_2	108.1(16)
C3_5	Ru1_1	Ge1_1	133.8(6)	C2_2	Si2B_2	Si1_2	127.8(12)
C2_5	Ru1_1	Ge1_1	139.9(4)	C9B_2	Si2B_2	Si1_2	109.6(16)
C5_5	Ru1_1	Ge1_1	111.5(4)	C1_2	Si2B_2	Si1_2	100.0(8)
Ge4_1	Ru1_1	Ge1_1	57.42(4)	C6_2	Si3B_2	C7B_2	93.7(19)
Ge8_1	Ru1_1	Ge1_1	58.21(4)	C6_2	Si3B_2	C5_2	109.6(11)
Ge7_1	Ru1_1	Ge1_1	99.76(4)	C7B_2	Si3B_2	C5_2	99.4(18)
Ge3_1	Ru1_1	Ge1_1	80.52(4)	C6_2	Si3B_2	Si1_2	124.8(10)
Si1_3	Ge1_1	Ge4_1	117.85(8)	C7B_2	Si3B_2	Si1_2	111.1(18)
Si1_3	Ge1_1	Ge8_1	117.51(9)	C5_2	Si3B_2	Si1_2	113.5(9)
Ge4_1	Ge1_1	Ge8_1	116.91(5)	Si2_3	Si1_3	Si4_3	112.4(2)
Si1_3	Ge1_1	Ge5_1	99.46(8)	Si2_3	Si1_3	Si3_3	103.71(19)
Ge4_1	Ge1_1	Ge5_1	120.65(6)	Si4_3	Si1_3	Si3_3	105.2(2)
Ge8_1	Ge1_1	Ge5_1	76.21(5)	Si2_3	Si1_3	Ge1_1	113.05(15)
Si1_3	Ge1_1	Ru1_1	141.33(9)	Si4_3	Si1_3	Ge1_1	111.26(16)
Ge4_1	Ge1_1	Ru1_1	59.27(4)	Si3_3	Si1_3	Ge1_1	110.68(17)
Ge8_1	Ge1_1	Ru1_1	58.99(4)	C1_3	Si2_3	C3_3	106.5(8)
Ge5_1	Ge1_1	Ru1_1	114.92(5)	C1_3	Si2_3	C2_3	106.0(8)
Si1_3	Ge1_1	Ge2_1	113.59(9)	C3_3	Si2_3	C2_3	108.0(9)
Ge4_1	Ge1_1	Ge2_1	64.20(5)	C1_3	Si2_3	Si1_3	115.4(6)
Ge8_1	Ge1_1	Ge2_1	115.62(5)	C3_3	Si2_3	Si1_3	110.6(5)
Ge5_1	Ge1_1	Ge2_1	58.92(4)	C2_3	Si2_3	Si1_3	110.1(7)
Ru1_1	Ge1_1	Ge2_1	99.60(5)	C4_3	Si3_3	C6_3	107.3(12)
Ge5_1	Ge2_1	Ge6_1	64.29(4)	C4_3	Si3_3	C5_3	106.4(8)
Ge5_1	Ge2_1	Ge3_1	99.18(5)	C6_3	Si3_3	C5_3	105.7(7)
Ge6_1	Ge2_1	Ge3_1	58.52(4)	C4_3	Si3_3	Si1_3	114.8(6)
Ge5_1	Ge2_1	Ge1_1	58.62(4)	C6_3	Si3_3	Si1_3	114.3(9)
Ge6_1	Ge2_1	Ge1_1	99.02(5)	C5_3	Si3_3	Si1_3	107.7(6)
Ge3_1	Ge2_1	Ge1_1	79.38(5)	C9_3	Si4_3	C7_3	106.8(8)
Ge5_1	Ge2_1	Ge4_1	111.26(6)	C9_3	Si4_3	C8_3	109.6(13)
Ge6_1	Ge2_1	Ge4_1	111.47(5)	C7_3	Si4_3	C8_3	105.1(10)
Ge3_1	Ge2_1	Ge4_1	55.15(4)	C9_3	Si4_3	Si1_3	114.0(8)
Ge1_1	Ge2_1	Ge4_1	54.68(4)	C7_3	Si4_3	Si1_3	110.5(6)
Si1_2	Ge3_1	Ge4_1	115.71(9)	C8_3	Si4_3	Si1_3	110.5(6)
Si1_2	Ge3_1	Ge7_1	119.59(9)	Si3_4	Si1_4	Si2_4	114.05(16)

Atom 1	Atom 2	Atom 3	Bond angle	Atom 1	Atom 2	Atom 3	Bond angle
Ge4_1	Ge3_1	Ge7_1	117.19(6)	Si3_4	Si1_4	Si4_4	111.14(17)
Si1_2	Ge3_1	Ge6_1	100.80(9)	Si2_4	Si1_4	Si4_4	111.95(17)
Ge4_1	Ge3_1	Ge6_1	120.97(5)	Si3_4	Si1_4	Ge9_1	105.60(14)
Ge7_1	Ge3_1	Ge6_1	74.98(4)	Si2_4	Si1_4	Ge9_1	106.44(14)
Si1_2	Ge3_1	Ru1_1	140.66(9)	Si4_4	Si1_4	Ge9_1	107.09(13)
Ge4_1	Ge3_1	Ru1_1	59.16(4)	C1_4	Si2_4	C2_4	108.4(8)
Ge7_1	Ge3_1	Ru1_1	59.61(4)	C1_4	Si2_4	C3_4	107.9(9)
Ge6_1	Ge3_1	Ru1_1	115.01(5)	C2_4	Si2_4	C3_4	110.3(10)
Si1_2	Ge3_1	Ge2_1	112.42(9)	C1_4	Si2_4	Si1_4	109.7(4)
Ge4_1	Ge3_1	Ge2_1	64.09(5)	C2_4	Si2_4	Si1_4	111.4(6)
Ge7_1	Ge3_1	Ge2_1	115.36(5)	C3_4	Si2_4	Si1_4	109.1(5)
Ge6_1	Ge3_1	Ge2_1	59.54(4)	C4_4	Si3_4	C5_4	109.2(7)
Ru1_1	Ge3_1	Ge2_1	99.93(5)	C4_4	Si3_4	C6_4	110.3(7)
Ge1_1	Ge4_1	Ge3_1	86.03(5)	C5_4	Si3_4	C6_4	108.4(8)
Ge1_1	Ge4_1	Ru1_1	63.31(4)	C4_4	Si3_4	Si1_4	110.5(4)
Ge3_1	Ge4_1	Ru1_1	62.92(4)	C5_4	Si3_4	Si1_4	109.5(5)
Ge1_1	Ge4_1	Ge2_1	61.12(4)	C6_4	Si3_4	Si1_4	109.0(5)
Ge3_1	Ge4_1	Ge2_1	60.76(4)	C8_4	Si4_4	C9_4	106.6(9)
Ru1_1	Ge4_1	Ge2_1	100.17(5)	C8_4	Si4_4	C7_4	109.5(12)
Ge9_1	Ge5_1	Ge1_1	95.98(5)	C9_4	Si4_4	C7_4	106.4(13)
Ge9_1	Ge5_1	Ge2_1	105.84(5)	C8_4	Si4_4	Si1_4	112.3(6)
Ge1_1	Ge5_1	Ge2_1	62.46(5)	C9_4	Si4_4	Si1_4	112.1(6)
Ge9_1	Ge5_1	Ge6_1	56.27(4)	C7_4	Si4_4	Si1_4	109.8(6)
Ge1_1	Ge5_1	Ge6_1	97.58(5)	C2_5	C1_5	C6_5	127.1(18)
Ge2_1	Ge5_1	Ge6_1	58.25(4)	C2_5	C1_5	C3_5	102.7(12)
Ge9_1	Ge6_1	Ge3_1	96.20(5)	C6_5	C1_5	C3_5	129.1(19)
Ge9_1	Ge6_1	Ge2_1	104.84(6)	C2_5	C1_5	Ru1_1	72.6(7)
Ge3_1	Ge6_1	Ge2_1	61.94(4)	C6_5	C1_5	Ru1_1	129.6(10)
Ge9_1	Ge6_1	Ge5_1	55.90(4)	C3_5	C1_5	Ru1_1	70.4(7)
Ge3_1	Ge6_1	Ge5_1	96.93(5)	C5_5	C2_5	C1_5	113.4(13)
Ge2_1	Ge6_1	Ge5_1	57.46(4)	C5_5	C2_5	C7_5	121.1(17)
Ge9_1	Ge7_1	Ge3_1	97.04(5)	C1_5	C2_5	C7_5	125.1(17)
Ge9_1	Ge7_1	Ru1_1	104.98(5)	C5_5	C2_5	Ru1_1	73.2(8)
Ge3_1	Ge7_1	Ru1_1	62.49(4)	C1_5	C2_5	Ru1_1	71.2(7)
Ge9_1	Ge7_1	Ge8_1	56.38(4)	C7_5	C2_5	Ru1_1	129.7(9)
Ge3_1	Ge7_1	Ge8_1	97.23(5)	C4_5	C3_5	C10_5	125(2)
Ru1_1	Ge7_1	Ge8_1	56.72(4)	C4_5	C3_5	C1_5	104.5(11)
Ge9_1	Ge8_1	Ge1_1	96.21(5)	C10_5	C3_5	C1_5	129(2)
Ge9_1	Ge8_1	Ru1_1	105.41(5)	C4_5	C3_5	Ru1_1	70.9(7)
Ge1_1	Ge8_1	Ru1_1	62.80(4)	C10_5	C3_5	Ru1_1	131.0(13)
Ge9_1	Ge8_1	Ge7_1	56.27(4)	C1_5	C3_5	Ru1_1	69.7(6)
Ge1_1	Ge8_1	Ge7_1	97.13(5)	C5_5	C4_5	C3_5	111.1(14)
Ru1_1	Ge8_1	Ge7_1	57.34(4)	C5_5	C4_5	C9_5	121.6(19)
Si1_4	Ge9_1	Ge5_1	119.33(8)	C3_5	C4_5	C9_5	126.5(18)
Si1_4	Ge9_1	Ge6_1	119.52(9)	C5_5	C4_5	Ru1_1	74.1(8)
Ge5_1	Ge9_1	Ge6_1	67.84(5)	C3_5	C4_5	Ru1_1	72.1(9)
Si1_4	Ge9_1	Ge7_1	126.74(8)	C9_5	C4_5	Ru1_1	129.3(12)
Ge5_1	Ge9_1	Ge7_1	113.78(5)	C4_5	C5_5	C2_5	108.1(14)
Ge6_1	Ge9_1	Ge7_1	76.95(5)	C4_5	C5_5	C8_5	124.0(19)
Si1_4	Ge9_1	Ge8_1	126.35(9)	C2_5	C5_5	C8_5	127.4(19)
Ge5_1	Ge9_1	Ge8_1	78.44(5)	C4_5	C5_5	Ru1_1	71.0(9)
Ge6_1	Ge9_1	Ge8_1	114.07(5)	C2_5	C5_5	Ru1_1	71.9(8)
Ge7_1	Ge9_1	Ge8_1	67.35(4)	C8_5	C5_5	Ru1_1	128.7(11)
Si3B_2	Si1_2	Si4B_2	113.2(6)				

[η^1-$Ge_9(Si^tBu_2H)_3$]$RuCp(PPh_3)_2$ (2)

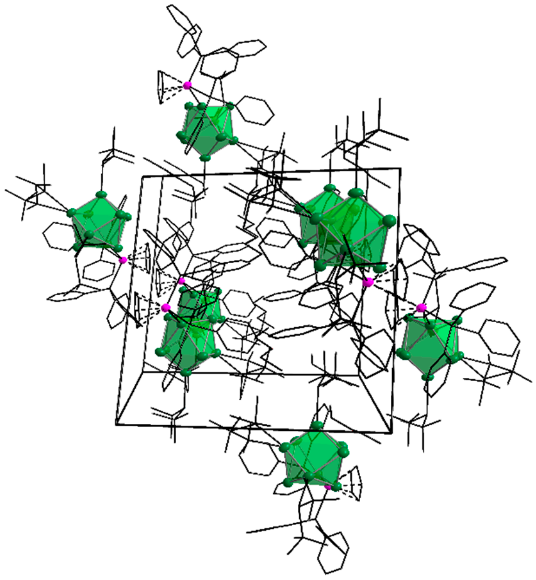

Figure S19. Unit cell of **2**. Cluster cores [Ge_9] are shown as green polyhedra, the silyl groups as well as the ligands of ruthenium are shown in wire and frame mode for simplicity. Germanium and ruthenium atoms are depicted in green and pink color.

Table S3. Bond lengths in [Å] in **2**. The numbers of the various residues are shown here using underscores. Residue 1 is the cluster core, residues 2, 3 and 4 are the silyl ligands, residue 5 is the bound ruthenium atom with its ligands and residue 6 contains the cocrystallised Et_2O molecules.

Atom 1	Atom 2	Bond length	Atom 1	Atom 2	Bond length
Ge1_1	Si1_3	2.400(3)	C7_5	C12_5	1.368(12)
Ge1_1	Ge2_1	2.4596(15)	C7_5	C8_5	1.404(11)
Ge1_1	Ge8_1	2.5369(13)	C8_5	C9_5	1.381(12)
Ge1_1	Ge4_1	2.5676(15)	C9_5	C10_5	1.372(13)
Ge1_1	Ge5_1	2.6727(15)	C10_5	C11_5	1.377(13)
Ge2_1	Si2_4	2.363(7)	C11_5	C12_5	1.369(12)
Ge2_1	Si1_4	2.439(4)	C13_5	C14_5	1.408(12)
Ge2_1	Ge3_1	2.4768(14)	C13_5	C18_5	1.410(12)
Ge2_1	Ge6_1	2.5800(13)	C14_5	C15_5	1.392(11)
Ge2_1	Ge5_1	2.6019(13)	C15_5	C16_5	1.382(13)
Ge3_1	Si1_2	2.399(3)	C16_5	C17_5	1.363(12)
Ge3_1	Ge7_1	2.5292(12)	C17_5	C18_5	1.389(11)
Ge3_1	Ge4_1	2.5789(15)	C19_5	C20_5	1.371(13)
Ge3_1	Ge6_1	2.6656(14)	C19_5	C24_5	1.414(11)
Ge4_1	Ge7_1	2.6447(13)	C20_5	C21_5	1.380(12)
Ge4_1	Ge8_1	2.6730(14)	C21_5	C22_5	1.377(12)
Ge5_1	Ge9_1	2.5656(12)	C22_5	C23_5	1.362(13)
Ge5_1	Ge6_1	2.8765(13)	C23_5	C24_5	1.380(12)
Ge6_1	Ge9_1	2.5415(12)	C25_5	C26_5	1.382(12)
Ge7_1	Ge9_1	2.5771(13)	C25_5	C30_5	1.405(13)
Ge7_1	Ge8_1	2.7584(13)	C26_5	C27_5	1.409(12)
Ge8_1	Ge9_1	2.5970(14)	C27_5	C28_5	1.340(13)
Ge9_1	Ru1_5	2.4822(10)	C28_5	C29_5	1.404(13)
Si1_2	C1_2	1.897(10)	C29_5	C30_5	1.389(12)
Si1_2	C5_2	1.936(9)	C31_5	C36_5	1.402(11)
C1_2	C3_2	1.499(12)	C31_5	C32_5	1.404(11)
C1_2	C4_2	1.521(14)	C32_5	C33_5	1.383(13)
C1_2	C2_2	1.528(12)	C33_5	C34_5	1.370(14)
C5_2	C6_2	1.519(14)	C34_5	C35_5	1.366(13)
C5_2	C8_2	1.521(13)	C35_5	C36_5	1.392(12)
C5_2	C7_2	1.550(13)	C37_5	C41_5	1.399(12)
Si1_3	C1_3	1.914(11)	C37_5	C38_5	1.412(12)
Si1_3	C5_3	1.915(9)	C38_5	C39_5	1.406(11)
C1_3	C3_3	1.538(13)	C39_5	C40_5	1.430(12)
C1_3	C2_3	1.550(12)	C40_5	C41_5	1.421(11)
C1_3	C4_3	1.552(13)	O1_6	C2_6	1.28(2)
C5_3	C7_3	1.484(14)	O1_6	C4_6	1.41(2)
C5_3	C8_3	1.526(12)	O2_6	C8_6	1.475(17)
C5_3	C6_3	1.536(13)	O2_6	C3_6	1.549(19)
Ru1_5	C37_5	2.212(8)	C1_6	C2_6	1.41(2)
Ru1_5	C38_5	2.217(8)	C3_6	C6_6	1.70(3)
Ru1_5	C41_5	2.219(9)	C4_6	C5_6	1.35(2)
Ru1_5	C39_5	2.224(8)	C7_6	C8_6	1.564(19)
Ru1_5	C40_5	2.245(9)	Si1_4	Si2_4	1.104(9)
Ru1_5	P2_5	2.320(3)	Si1_4	C4_4	1.814(11)
Ru1_5	P1_5	2.334(2)	Si1_4	C3_4	1.941(12)
P1_5	C1_5	1.845(9)	Si1_4	C9_4	2.03(2)
P1_5	C7_5	1.853(9)	Si2_4	C4_4	1.815(12)
P1_5	C13_5	1.861(8)	Si2_4	C3_4	1.982(14)
P2_5	C25_5	1.851(9)	Si2_4	C7_4	2.103(17)
P2_5	C31_5	1.853(8)	C1_4	C4_4	1.460(15)
P2_5	C19_5	1.861(9)	C2_4	C3_4	1.477(15)
C1_5	C2_5	1.359(13)	C3_4	C6_4	1.530(17)
C1_5	C6_5	1.399(11)	C3_4	C8_4	1.535(15)
C2_5	C3_5	1.422(14)	C4_4	C5_4	1.469(13)
C3_5	C4_5	1.368(15)	C4_4	C7_4	1.730(19)
C4_5	C5_5	1.368(16)	C4_4	C9_4	1.90(3)
C5_5	C6_5	1.376(14)			

Table S4. Bond angles in [°] in **2**. The numbers of the various residues are shown here using underscores. Residue 1 is the cluster core, residues 2, 3 and 4 are the silyl ligands, residue 5 is the bound ruthenium atom with its ligands and residue 6 contains the cocrystallised Et_2O molecules.

Atom 1	Atom 2	Atom 3	Bond angle	Atom 1	Atom 2	Atom 3	Bond angle
Si1_3	Ge1_1	Ge2_1	125.19(8)	P2_5	Ru1_5	Ge9_1	91.45(6)
Si1_3	Ge1_1	Ge8_1	120.23(8)	P1_5	Ru1_5	Ge9_1	97.02(6)
Ge2_1	Ge1_1	Ge8_1	108.67(5)	C1_5	P1_5	C7_5	97.6(4)
Si1_3	Ge1_1	Ge4_1	127.71(9)	C1_5	P1_5	C13_5	104.5(4)
Ge2_1	Ge1_1	Ge4_1	94.76(5)	C7_5	P1_5	C13_5	99.4(4)
Ge8_1	Ge1_1	Ge4_1	63.15(4)	C1_5	P1_5	Ru1_5	126.0(3)
Si1_3	Ge1_1	Ge5_1	113.89(8)	C7_5	P1_5	Ru1_5	116.9(3)
Ge2_1	Ge1_1	Ge5_1	60.75(4)	C13_5	P1_5	Ru1_5	108.7(3)
Ge8_1	Ge1_1	Ge5_1	69.87(4)	C25_5	P2_5	C31_5	99.9(4)
Ge4_1	Ge1_1	Ge5_1	114.92(4)	C25_5	P2_5	C19_5	101.7(4)
Si2_4	Ge2_1	Si1_4	26.5(2)	C31_5	P2_5	C19_5	99.4(4)
Si2_4	Ge2_1	Ge1_1	109.1(2)	C25_5	P2_5	Ru1_5	112.6(3)
Si1_4	Ge2_1	Ge1_1	133.96(13)	C31_5	P2_5	Ru1_5	125.7(3)
Si2_4	Ge2_1	Ge3_1	140.0(2)	C19_5	P2_5	Ru1_5	114.0(3)
Si1_4	Ge2_1	Ge3_1	120.60(12)	C2_5	C1_5	C6_5	119.3(9)
Ge1_1	Ge2_1	Ge3_1	87.65(5)	C2_5	C1_5	P1_5	124.2(7)
Si2_4	Ge2_1	Ge6_1	135.0(2)	C6_5	C1_5	P1_5	116.4(7)
Si1_4	Ge2_1	Ge6_1	115.47(12)	C1_5	C2_5	C3_5	120.5(10)
Ge1_1	Ge2_1	Ge6_1	109.49(4)	C4_5	C3_5	C2_5	118.8(12)
Ge3_1	Ge2_1	Ge6_1	63.59(4)	C5_5	C4_5	C3_5	120.8(11)
Si2_4	Ge2_1	Ge5_1	111.6(2)	C4_5	C5_5	C6_5	120.4(11)
Si1_4	Ge2_1	Ge5_1	126.35(12)	C5_5	C6_5	C1_5	120.2(11)
Ge1_1	Ge2_1	Ge5_1	63.67(4)	C12_5	C7_5	C8_5	118.0(8)
Ge3_1	Ge2_1	Ge5_1	108.39(4)	C12_5	C7_5	P1_5	122.9(7)
Ge6_1	Ge2_1	Ge5_1	67.43(4)	C8_5	C7_5	P1_5	119.1(7)
Si1_2	Ge3_1	Ge2_1	127.71(8)	C9_5	C8_5	C7_5	120.0(9)
Si1_2	Ge3_1	Ge7_1	114.60(7)	C10_5	C9_5	C8_5	120.8(9)
Ge2_1	Ge3_1	Ge7_1	109.27(4)	C9_5	C10_5	C11_5	119.0(10)
Si1_2	Ge3_1	Ge4_1	130.82(8)	C12_5	C11_5	C10_5	120.5(10)
Ge2_1	Ge3_1	Ge4_1	94.06(5)	C7_5	C12_5	C11_5	121.6(9)
Ge7_1	Ge3_1	Ge4_1	62.35(4)	C14_5	C13_5	C18_5	117.4(7)
Si1_2	Ge3_1	Ge6_1	108.29(8)	C14_5	C13_5	P1_5	121.5(7)
Ge2_1	Ge3_1	Ge6_1	60.09(4)	C18_5	C13_5	P1_5	120.6(6)
Ge7_1	Ge3_1	Ge6_1	72.35(4)	C15_5	C14_5	C13_5	120.7(9)
Ge4_1	Ge3_1	Ge6_1	115.53(4)	C16_5	C15_5	C14_5	120.4(9)
Ge1_1	Ge4_1	Ge3_1	83.24(5)	C17_5	C16_5	C15_5	119.8(8)
Ge1_1	Ge4_1	Ge7_1	99.53(5)	C16_5	C17_5	C18_5	121.2(9)
Ge3_1	Ge4_1	Ge7_1	57.90(4)	C17_5	C18_5	C13_5	120.5(8)
Ge1_1	Ge4_1	Ge8_1	57.86(4)	C20_5	C19_5	C24_5	118.1(9)
Ge3_1	Ge4_1	Ge8_1	98.58(5)	C20_5	C19_5	P2_5	122.4(7)
Ge7_1	Ge4_1	Ge8_1	62.49(4)	C24_5	C19_5	P2_5	119.5(8)
Ge9_1	Ge5_1	Ge2_1	101.77(4)	C19_5	C20_5	C21_5	121.3(9)
Ge9_1	Ge5_1	Ge1_1	100.63(5)	C22_5	C21_5	C20_5	120.4(10)
Ge2_1	Ge5_1	Ge1_1	55.57(4)	C23_5	C22_5	C21_5	119.2(9)
Ge9_1	Ge5_1	Ge6_1	55.33(3)	C22_5	C23_5	C24_5	121.4(9)
Ge2_1	Ge5_1	Ge6_1	55.92(3)	C23_5	C24_5	C19_5	119.6(9)
Ge1_1	Ge5_1	Ge6_1	95.68(4)	C26_5	C25_5	C30_5	118.0(8)
Ge9_1	Ge6_1	Ge2_1	103.04(4)	C26_5	C25_5	P2_5	123.7(7)
Ge9_1	Ge6_1	Ge3_1	99.55(5)	C30_5	C25_5	P2_5	118.3(7)
Ge2_1	Ge6_1	Ge3_1	56.32(4)	C25_5	C26_5	C27_5	119.9(9)
Ge9_1	Ge6_1	Ge5_1	56.12(3)	C28_5	C27_5	C26_5	121.7(9)
Ge2_1	Ge6_1	Ge5_1	56.64(3)	C27_5	C28_5	C29_5	120.0(9)
Ge3_1	Ge6_1	Ge5_1	95.95(4)	C30_5	C29_5	C28_5	118.8(10)
Ge3_1	Ge7_1	Ge9_1	102.29(4)	C29_5	C30_5	C25_5	121.6(9)
Ge3_1	Ge7_1	Ge4_1	59.74(4)	C36_5	C31_5	C32_5	119.4(8)
Ge9_1	Ge7_1	Ge4_1	110.29(4)	C36_5	C31_5	P2_5	118.8(6)
Ge3_1	Ge7_1	Ge8_1	97.60(4)	C32_5	C31_5	P2_5	121.7(7)
Ge9_1	Ge7_1	Ge8_1	58.13(4)	C33_5	C32_5	C31_5	119.0(9)
Ge4_1	Ge7_1	Ge8_1	59.26(3)	C34_5	C33_5	C32_5	120.8(9)
Ge1_1	Ge8_1	Ge9_1	103.49(5)	C35_5	C34_5	C33_5	121.1(10)
Ge1_1	Ge8_1	Ge4_1	58.98(4)	C34_5	C35_5	C36_5	119.7(10)
Ge9_1	Ge8_1	Ge4_1	108.80(4)	C35_5	C36_5	C31_5	119.9(9)
Ge1_1	Ge8_1	Ge7_1	97.35(4)	C41_5	C37_5	C38_5	108.4(8)
Ge9_1	Ge8_1	Ge7_1	57.43(3)	C41_5	C37_5	Ru1_5	71.9(5)
Ge4_1	Ge8_1	Ge7_1	58.25(3)	C38_5	C37_5	Ru1_5	71.6(5)
Ru1_5	Ge9_1	Ge6_1	137.74(5)	C39_5	C38_5	C37_5	107.5(8)
Ru1_5	Ge9_1	Ge5_1	127.67(5)	C39_5	C38_5	Ru1_5	71.8(4)
Ge6_1	Ge9_1	Ge5_1	68.56(4)	C37_5	C38_5	Ru1_5	71.2(4)
Ru1_5	Ge9_1	Ge7_1	122.53(4)	C38_5	C39_5	C40_5	109.0(8)
Ge6_1	Ge9_1	Ge7_1	73.65(4)	C38_5	C39_5	Ru1_5	71.3(5)
Ge5_1	Ge9_1	Ge7_1	107.06(4)	C40_5	C39_5	Ru1_5	72.1(5)
Ru1_5	Ge9_1	Ge8_1	115.09(4)	C41_5	C40_5	C39_5	106.0(7)

Atom 1	Atom 2	Atom 3	Bond angle	Atom 1	Atom 2	Atom 3	Bond angle
Ge6_1	Ge9_1	Ge8_1	107.10(4)	C41_5	C40_5	Ru1_5	70.5(5)
Ge5_1	Ge9_1	Ge8_1	70.65(4)	C39_5	C40_5	Ru1_5	70.5(5)
Ge7_1	Ge9_1	Ge8_1	64.43(4)	C37_5	C41_5	C40_5	109.1(8)
C1_2	Si1_2	C5_2	116.6(4)	C37_5	C41_5	Ru1_5	71.3(5)
C1_2	Si1_2	Ge3_1	105.6(3)	C40_5	C41_5	Ru1_5	72.4(5)
C5_2	Si1_2	Ge3_1	116.1(3)	C2_6	O1_6	C4_6	120(2)
C3_2	C1_2	C4_2	108.5(9)	C8_6	O2_6	C3_6	106.5(14)
C3_2	C1_2	C2_2	108.6(8)	O1_6	C2_6	C1_6	125(2)
C4_2	C1_2	C2_2	105.1(9)	O2_6	C3_6	C6_6	96.2(18)
C3_2	C1_2	Si1_2	108.6(7)	C5_6	C4_6	O1_6	113(2)
C4_2	C1_2	Si1_2	113.9(6)	O2_6	C8_6	C7_6	105.9(13)
C2_2	C1_2	Si1_2	112.0(8)	Si2_4	Si1_4	C4_4	72.3(6)
C6_2	C5_2	C8_2	108.4(8)	Si2_4	Si1_4	C3_4	75.7(7)
C6_2	C5_2	C7_2	111.1(8)	C4_4	Si1_4	C3_4	120.3(6)
C8_2	C5_2	C7_2	106.1(9)	Si2_4	Si1_4	C9_4	130.4(9)
C6_2	C5_2	Si1_2	112.2(7)	C4_4	Si1_4	C9_4	58.8(8)
C8_2	C5_2	Si1_2	111.2(6)	C3_4	Si1_4	C9_4	135.6(9)
C7_2	C5_2	Si1_2	107.7(6)	Si2_4	Si1_4	Ge2_1	72.9(4)
C1_3	Si1_3	C5_3	118.6(4)	C4_4	Si1_4	Ge2_1	108.4(4)
C1_3	Si1_3	Ge1_1	107.0(3)	C3_4	Si1_4	Ge2_1	108.4(4)
C5_3	Si1_3	Ge1_1	113.7(3)	C9_4	Si1_4	Ge2_1	113.5(9)
C3_3	C1_3	C2_3	107.8(8)	Si1_4	Si2_4	C4_4	72.3(7)
C3_3	C1_3	C4_3	108.7(9)	Si1_4	Si2_4	C3_4	71.6(7)
C2_3	C1_3	C4_3	107.1(9)	C4_4	Si2_4	C3_4	118.1(7)
C3_3	C1_3	Si1_3	106.8(7)	Si1_4	Si2_4	C7_4	123.3(7)
C2_3	C1_3	Si1_3	111.9(7)	C4_4	Si2_4	C7_4	51.8(6)
C4_3	C1_3	Si1_3	114.3(6)	C3_4	Si2_4	C7_4	124.6(7)
C7_3	C5_3	C8_3	108.3(8)	Si1_4	Si2_4	Ge2_1	80.5(4)
C7_3	C5_3	C6_3	109.5(8)	C4_4	Si2_4	Ge2_1	111.6(5)
C8_3	C5_3	C6_3	108.7(9)	C3_4	Si2_4	Ge2_1	109.9(4)
C7_3	C5_3	Si1_3	108.2(7)	C7_4	Si2_4	Ge2_1	124.6(6)
C8_3	C5_3	Si1_3	109.7(6)	C2_4	C3_4	C6_4	108.1(10)
C6_3	C5_3	Si1_3	112.4(6)	C2_4	C3_4	C8_4	108.3(12)
C37_5	Ru1_5	C38_5	37.2(3)	C6_4	C3_4	C8_4	110.1(12)
C37_5	Ru1_5	C41_5	36.8(3)	C2_4	C3_4	Si1_4	99.8(9)
C38_5	Ru1_5	C41_5	61.9(3)	C6_4	C3_4	Si1_4	119.0(9)
C37_5	Ru1_5	C39_5	61.7(3)	C8_4	C3_4	Si1_4	110.6(8)
C38_5	Ru1_5	C39_5	36.9(3)	C2_4	C3_4	Si2_4	126.0(9)
C41_5	Ru1_5	C39_5	61.6(3)	C6_4	C3_4	Si2_4	89.9(9)
C37_5	Ru1_5	C40_5	62.1(3)	C8_4	C3_4	Si2_4	112.3(8)
C38_5	Ru1_5	C40_5	62.3(3)	Si1_4	C3_4	Si2_4	32.7(3)
C41_5	Ru1_5	C40_5	37.1(3)	C1_4	C4_4	C5_4	114.1(9)
C39_5	Ru1_5	C40_5	37.3(3)	C1_4	C4_4	C7_4	97.8(11)
C37_5	Ru1_5	P2_5	145.4(2)	C5_4	C4_4	C7_4	95.8(10)
C38_5	Ru1_5	P2_5	108.3(2)	C1_4	C4_4	Si1_4	117.3(8)
C41_5	Ru1_5	P2_5	143.6(2)	C5_4	C4_4	Si1_4	118.8(9)
C39_5	Ru1_5	P2_5	89.2(2)	C7_4	C4_4	Si1_4	107.7(8)
C40_5	Ru1_5	P2_5	106.5(2)	C1_4	C4_4	Si2_4	117.2(8)
C37_5	Ru1_5	P1_5	86.9(2)	C5_4	C4_4	Si2_4	128.4(9)
C38_5	Ru1_5	P1_5	96.9(2)	C7_4	C4_4	Si2_4	72.7(8)
C41_5	Ru1_5	P1_5	113.3(2)	Si1_4	C4_4	Si2_4	35.4(3)
C39_5	Ru1_5	P1_5	132.7(2)	C1_4	C4_4	C9_4	86.1(12)
C40_5	Ru1_5	P1_5	148.5(2)	C5_4	C4_4	C9_4	86.8(11)
P2_5	Ru1_5	P1_5	102.43(9)	C7_4	C4_4	C9_4	174.0(11)
C37_5	Ru1_5	Ge9_1	120.7(2)	Si1_4	C4_4	C9_4	66.3(8)
C38_5	Ru1_5	Ge9_1	152.7(2)	Si2_4	C4_4	C9_4	101.4(9)
C41_5	Ru1_5	Ge9_1	91.1(2)	C4_4	C7_4	Si2_4	55.5(6)
C39_5	Ru1_5	Ge9_1	128.7(2)	C4_4	C9_4	Si1_4	54.9(7)
C40_5	Ru1_5	Ge9_1	94.6(2)				

[Hyp_3Ge_9][$RuCp(PPh_3)_2(MeCN)$] (3b)

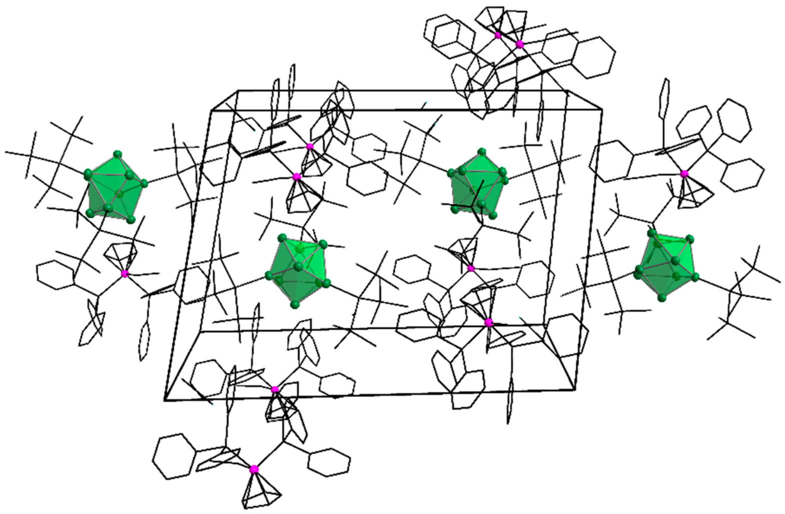

Figure S20. Unit cell of **3b**. Cluster cores [Ge_9] are shown as green polyhedra, whereby the hypersilyl groups are depicted in wire and frame for simplicity. The ruthenium counter ion complex is also simplified for clarity reasons. Germanium and ruthenium atoms are depicted in green and pink color, respectively.

Table S5. Bond lengths in [Å] in **3b**. The numbers of the various residues are shown here using underscores. Residue 1 is the cluster core with its silyl groups, residue 2 is the ruthenium counter ion complex and residue 3 contains the cocrystallised MeCN molecules.

Atom 1	Atom 2	Bond length	Atom 1	Atom 2	Bond length
Ge3_1	Si9_1	2.3771(10)	Ru1_2	C4_2	2.218(3)
Ge3_1	Ge6_1	2.5401(5)	Ru1_2	C1_2	2.221(3)
Ge3_1	Ge2_1	2.5413(5)	Ru1_2	C3_2	2.228(3)
Ge3_1	Ge7_1	2.5432(5)	Ru1_2	C2_2	2.237(3)
Ge3_1	Ge4_1	2.5559(5)	Ru1_2	P2_2	2.3356(9)
Ge4_1	Ge1_1	2.5324(5)	Ru1_2	P1_2	2.3446(8)
Ge4_1	Ge7_1	2.6584(5)	P1_2	C18_2	1.825(4)
Ge4_1	Ge8_1	2.7579(5)	P1_2	C12_2	1.835(3)
Ge5_1	Ge1_1	2.5294(5)	P1_2	C6_2	1.838(3)
Ge5_1	Ge9_1	2.5425(5)	P2_2	C24_2	1.836(3)
Ge5_1	Ge6_1	2.6485(5)	P2_2	C36_2	1.837(3)
Ge5_1	Ge2_1	2.7568(5)	P2_2	C30_2	1.850(3)
Ge2_1	Ge1_1	2.5409(5)	N1_2	C42_2	1.148(4)
Ge2_1	Ge6_1	2.6556(5)	C1_2	C2_2	1.419(5)
Ge1_1	Si5_1	2.3763(10)	C1_2	C5_2	1.429(5)
Ge1_1	Ge8_1	2.5212(5)	C2_2	C3_2	1.442(6)
Ge6_1	Ge9_1	2.5305(5)	C3_2	C4_2	1.407(6)
Ge7_1	Ge9_1	2.5442(5)	C4_2	C5_2	1.436(6)
Ge7_1	Ge8_1	2.6429(5)	C6_2	C7_2	1.393(5)
Ge8_1	Ge9_1	2.5575(5)	C6_2	C11_2	1.402(5)
Ge9_1	Si1_1	2.3697(9)	C7_2	C8_2	1.392(5)
Si1_1	Si3_1	2.3438(15)	C8_2	C9_2	1.393(6)
Si1_1	Si4_1	2.3463(13)	C9_2	C10_2	1.388(6)
Si1_1	Si2_1	2.3580(15)	C10_2	C11_2	1.381(5)
Si2_1	C3_1	1.860(5)	C12_2	C13_2	1.390(5)
Si2_1	C2_1	1.877(4)	C12_2	C17_2	1.391(5)
Si2_1	C1_1	1.891(4)	C13_2	C14_2	1.395(5)
Si3_1	C4_1	1.872(5)	C14_2	C15_2	1.374(6)
Si3_1	C6_1	1.877(5)	C15_2	C16_2	1.382(7)
Si3_1	C5_1	1.881(6)	C16_2	C17_2	1.387(6)
Si4_1	C7_1	1.880(4)	C18_2	C23_2	1.403(5)
Si4_1	C8_1	1.885(4)	C18_2	C19_2	1.413(5)
Si4_1	C9_1	1.889(4)	C19_2	C20_2	1.388(5)
Si5_1	Si8_1	2.3459(13)	C20_2	C21_2	1.372(5)
Si5_1	Si6_1	2.3504(15)	C21_2	C22_2	1.386(6)
Si5_1	Si7_1	2.3545(14)	C22_2	C23_2	1.397(5)
Si6_1	C11_1	1.872(5)	C24_2	C29_2	1.394(5)
Si6_1	C10_1	1.873(4)	C24_2	C25_2	1.402(5)
Si6_1	C12_1	1.880(5)	C25_2	C26_2	1.384(5)
Si7_1	C13_1	1.861(5)	C26_2	C27_2	1.385(6)
Si7_1	C15_1	1.864(6)	C27_2	C28_2	1.374(7)
Si7_1	C14_1	1.879(5)	C28_2	C29_2	1.391(5)
Si8_1	C16_1	1.871(5)	C30_2	C35_2	1.377(5)
Si8_1	C17_1	1.877(4)	C30_2	C31_2	1.387(5)
Si8_1	C18_1	1.883(4)	C31_2	C32_2	1.400(5)
Si9_1	Si11_1	2.3459(13)	C32_2	C33_2	1.373(6)
Si9_1	Si10_1	2.3472(13)	C33_2	C34_2	1.369(6)
Si9_1	Si12_1	2.3491(13)	C34_2	C35_2	1.400(6)
Si10_1	C19_1	1.857(5)	C36_2	C41_2	1.397(4)
Si10_1	C20_1	1.875(4)	C36_2	C37_2	1.398(5)
Si10_1	C21_1	1.883(5)	C37_2	C38_2	1.394(5)
Si11_1	C22_1	1.873(5)	C38_2	C39_2	1.381(5)
Si11_1	C23_1	1.876(4)	C39_2	C40_2	1.389(5)
Si11_1	C24_1	1.887(5)	C40_2	C41_2	1.392(5)
Si12_1	C27_1	1.875(5)	C42_2	C43_2	1.452(5)
Si12_1	C26_1	1.879(5)	N1_3	C1_3	1.156(10)
Si12_1	C25_1	1.887(4)	N2_3	C3_3	1.118(8)
Ru1_2	N1_2	2.049(3)	C1_3	C2_3	1.427(10)
Ru1_2	C5_2	2.189(3)	C3_3	C4_3	1.431(10)

Table S6. Bond angles in [°] in **3b**. The numbers of the various residues are shown here using underscores. Residue 1 is the cluster core with its silyl groups, residue 2 is the ruthenium counter ion complex and residue 3 contains the cocrystallised MeCN molecules.

Atom 1	Atom 2	Atom 3	Bond angle	Atom 1	Atom 2	Atom 3	Bond angle
Si9_1	Ge3_1	Ge6_1	121.59(3)	C19_1	Si10_1	Si9_1	110.00(14)
Si9_1	Ge3_1	Ge2_1	114.92(3)	C20_1	Si10_1	Si9_1	111.27(15)
Ge6_1	Ge3_1	Ge2_1	63.016(15)	C21_1	Si10_1	Si9_1	109.84(15)
Si9_1	Ge3_1	Ge7_1	125.26(3)	C22_1	Si11_1	C23_1	107.1(2)
Ge6_1	Ge3_1	Ge7_1	93.198(17)	C22_1	Si11_1	C24_1	110.3(3)
Ge2_1	Ge3_1	Ge7_1	118.492(18)	C23_1	Si11_1	C24_1	108.0(2)
Si9_1	Ge3_1	Ge4_1	117.81(3)	C22_1	Si11_1	Si9_1	109.21(15)
Ge6_1	Ge3_1	Ge4_1	118.680(18)	C23_1	Si11_1	Si9_1	112.54(16)
Ge2_1	Ge3_1	Ge4_1	79.948(16)	C24_1	Si11_1	Si9_1	109.65(16)
Ge7_1	Ge3_1	Ge4_1	62.845(14)	C27_1	Si12_1	C26_1	107.0(2)
Ge1_1	Ge4_1	Ge3_1	93.248(16)	C27_1	Si12_1	C25_1	107.7(2)
Ge1_1	Ge4_1	Ge7_1	102.400(16)	C26_1	Si12_1	C25_1	109.5(2)
Ge3_1	Ge4_1	Ge7_1	58.343(14)	C27_1	Si12_1	Si9_1	111.73(15)
Ge1_1	Ge4_1	Ge8_1	56.731(14)	C26_1	Si12_1	Si9_1	111.21(16)
Ge3_1	Ge4_1	Ge8_1	97.161(15)	C25_1	Si12_1	Si9_1	109.57(15)
Ge7_1	Ge4_1	Ge8_1	58.377(13)	N1_2	Ru1_2	C5_2	154.88(13)
Ge1_1	Ge5_1	Ge9_1	93.648(17)	N1_2	Ru1_2	C4_2	118.91(13)
Ge1_1	Ge5_1	Ge6_1	103.194(17)	C5_2	Ru1_2	C4_2	38.02(14)
Ge9_1	Ge5_1	Ge6_1	58.307(14)	N1_2	Ru1_2	C1_2	134.53(13)
Ge1_1	Ge5_1	Ge2_1	57.265(14)	C5_2	Ru1_2	C1_2	37.79(14)
Ge9_1	Ge5_1	Ge2_1	97.641(17)	C4_2	Ru1_2	C1_2	62.57(15)
Ge6_1	Ge5_1	Ge2_1	58.812(14)	N1_2	Ru1_2	C3_2	92.16(13)
Ge1_1	Ge2_1	Ge3_1	93.395(17)	C5_2	Ru1_2	C3_2	62.92(14)
Ge1_1	Ge2_1	Ge6_1	102.678(17)	C4_2	Ru1_2	C3_2	36.90(15)
Ge3_1	Ge2_1	Ge6_1	58.471(14)	C1_2	Ru1_2	C3_2	62.32(14)
Ge1_1	Ge2_1	Ge5_1	56.863(14)	N1_2	Ru1_2	C2_2	99.74(13)
Ge3_1	Ge2_1	Ge5_1	97.442(16)	C5_2	Ru1_2	C2_2	63.06(14)
Ge6_1	Ge2_1	Ge5_1	58.560(13)	C4_2	Ru1_2	C2_2	62.48(15)
Si5_1	Ge1_1	Ge8_1	119.71(3)	C1_2	Ru1_2	C2_2	37.11(13)
Si5_1	Ge1_1	Ge5_1	119.62(3)	C3_2	Ru1_2	C2_2	37.68(14)
Ge8_1	Ge1_1	Ge5_1	80.690(16)	N1_2	Ru1_2	P2_2	87.05(8)
Si5_1	Ge1_1	Ge4_1	124.77(3)	C5_2	Ru1_2	P2_2	100.21(11)
Ge8_1	Ge1_1	Ge4_1	66.149(15)	C4_2	Ru1_2	P2_2	89.96(11)
Ge5_1	Ge1_1	Ge4_1	115.527(18)	C1_2	Ru1_2	P2_2	136.89(10)
Si5_1	Ge1_1	Ge2_1	124.89(3)	C3_2	Ru1_2	P2_2	115.08(11)
Ge8_1	Ge1_1	Ge2_1	115.301(18)	C2_2	Ru1_2	P2_2	151.53(11)
Ge5_1	Ge1_1	Ge2_1	65.872(15)	N1_2	Ru1_2	P1_2	98.49(8)
Ge4_1	Ge1_1	Ge2_1	80.401(16)	C5_2	Ru1_2	P1_2	104.02(10)
Ge9_1	Ge6_1	Ge3_1	84.948(16)	C4_2	Ru1_2	P1_2	142.02(11)
Ge9_1	Ge6_1	Ge5_1	58.749(14)	C1_2	Ru1_2	P1_2	87.19(10)
Ge3_1	Ge6_1	Ge5_1	100.299(16)	C3_2	Ru1_2	P1_2	144.93(11)
Ge9_1	Ge6_1	Ge2_1	100.599(16)	C2_2	Ru1_2	P1_2	107.33(11)
Ge3_1	Ge6_1	Ge2_1	58.513(14)	P2_2	Ru1_2	P1_2	98.83(3)
Ge5_1	Ge6_1	Ge2_1	62.629(14)	C18_2	P1_2	C12_2	105.21(16)
Ge3_1	Ge7_1	Ge9_1	84.604(16)	C18_2	P1_2	C6_2	104.73(15)
Ge3_1	Ge7_1	Ge8_1	100.473(16)	C12_2	P1_2	C6_2	96.55(15)
Ge9_1	Ge7_1	Ge8_1	59.046(14)	C18_2	P1_2	Ru1_2	109.81(11)
Ge3_1	Ge7_1	Ge4_1	58.811(14)	C12_2	P1_2	Ru1_2	125.96(12)
Ge9_1	Ge7_1	Ge4_1	100.751(17)	C6_2	P1_2	Ru1_2	112.22(11)
Ge8_1	Ge7_1	Ge4_1	62.696(14)	C24_2	P2_2	C36_2	103.51(15)
Ge1_1	Ge8_1	Ge9_1	93.481(16)	C24_2	P2_2	C30_2	103.09(16)
Ge1_1	Ge8_1	Ge7_1	103.142(17)	C36_2	P2_2	C30_2	101.66(15)
Ge9_1	Ge8_1	Ge7_1	58.553(14)	C24_2	P2_2	Ru1_2	112.57(12)
Ge1_1	Ge8_1	Ge4_1	57.120(14)	C36_2	P2_2	Ru1_2	121.77(11)
Ge9_1	Ge8_1	Ge4_1	97.808(16)	C30_2	P2_2	Ru1_2	112.14(11)
Ge7_1	Ge8_1	Ge4_1	58.927(13)	C42_2	N1_2	Ru1_2	168.0(3)
Si1_1	Ge9_1	Ge6_1	119.20(3)	C2_2	C1_2	C5_2	108.8(3)
Si1_1	Ge9_1	Ge5_1	117.40(3)	C2_2	C1_2	Ru1_2	72.03(19)
Ge6_1	Ge9_1	Ge5_1	62.944(15)	C5_2	C1_2	Ru1_2	69.89(19)
Si1_1	Ge9_1	Ge7_1	123.70(3)	C1_2	C2_2	C3_2	107.2(3)
Ge6_1	Ge9_1	Ge7_1	93.403(17)	C1_2	C2_2	Ru1_2	70.86(19)
Ge5_1	Ge9_1	Ge7_1	118.206(17)	C3_2	C2_2	Ru1_2	70.82(19)
Si1_1	Ge9_1	Ge8_1	121.20(3)	C4_2	C3_2	C2_2	108.4(3)
Ge6_1	Ge9_1	Ge8_1	118.398(17)	C4_2	C3_2	Ru1_2	71.18(19)
Ge5_1	Ge9_1	Ge8_1	79.750(16)	C2_2	C3_2	Ru1_2	71.50(19)
Ge7_1	Ge9_1	Ge8_1	62.401(15)	C3_2	C4_2	C5_2	108.4(4)
Si3_1	Si1_1	Si4_1	112.51(6)	C3_2	C4_2	Ru1_2	71.9(2)
Si3_1	Si1_1	Si2_1	113.00(6)	C5_2	C4_2	Ru1_2	69.91(19)
Si4_1	Si1_1	Si2_1	107.45(5)	C1_2	C5_2	C4_2	107.2(3)
Si3_1	Si1_1	Ge9_1	106.23(5)	C1_2	C5_2	Ru1_2	72.32(19)
Si4_1	Si1_1	Ge9_1	106.46(4)	C4_2	C5_2	Ru1_2	72.1(2)
Si2_1	Si1_1	Ge9_1	111.04(5)	C7_2	C6_2	C11_2	118.4(3)

Atom 1	Atom 2	Atom 3	Bond angle	Atom 1	Atom 2	Atom 3	Bond angle
C3_1	Si2_1	C2_1	108.6(2)	C7_2	C6_2	P1_2	123.7(3)
C3_1	Si2_1	C1_1	108.9(2)	C11_2	C6_2	P1_2	117.7(3)
C2_1	Si2_1	C1_1	109.3(2)	C8_2	C7_2	C6_2	120.3(4)
C3_1	Si2_1	Si1_1	111.57(15)	C7_2	C8_2	C9_2	120.5(4)
C2_1	Si2_1	Si1_1	110.47(17)	C10_2	C9_2	C8_2	119.6(4)
C1_1	Si2_1	Si1_1	107.87(16)	C11_2	C10_2	C9_2	119.8(4)
C4_1	Si3_1	C6_1	108.4(3)	C10_2	C11_2	C6_2	121.4(3)
C4_1	Si3_1	C5_1	108.4(3)	C13_2	C12_2	C17_2	118.6(3)
C6_1	Si3_1	C5_1	108.7(4)	C13_2	C12_2	P1_2	119.7(3)
C4_1	Si3_1	Si1_1	110.94(19)	C17_2	C12_2	P1_2	121.2(3)
C6_1	Si3_1	Si1_1	111.34(19)	C12_2	C13_2	C14_2	120.8(4)
C5_1	Si3_1	Si1_1	108.92(19)	C15_2	C14_2	C13_2	120.0(4)
C7_1	Si4_1	C8_1	108.2(2)	C14_2	C15_2	C16_2	119.7(4)
C7_1	Si4_1	C9_1	107.8(2)	C15_2	C16_2	C17_2	120.6(4)
C8_1	Si4_1	C9_1	109.8(2)	C16_2	C17_2	C12_2	120.3(4)
C7_1	Si4_1	Si1_1	111.34(15)	C23_2	C18_2	C19_2	117.8(3)
C8_1	Si4_1	Si1_1	109.35(14)	C23_2	C18_2	P1_2	121.8(3)
C9_1	Si4_1	Si1_1	110.33(15)	C19_2	C18_2	P1_2	119.8(3)
Si8_1	Si5_1	Si6_1	113.11(5)	C20_2	C19_2	C18_2	120.6(3)
Si8_1	Si5_1	Si7_1	110.46(5)	C21_2	C20_2	C19_2	120.8(4)
Si6_1	Si5_1	Si7_1	109.88(6)	C20_2	C21_2	C22_2	120.0(3)
Si8_1	Si5_1	Ge1_1	105.61(4)	C21_2	C22_2	C23_2	120.2(3)
Si6_1	Si5_1	Ge1_1	108.27(5)	C22_2	C23_2	C18_2	120.7(3)
Si7_1	Si5_1	Ge1_1	109.36(5)	C29_2	C24_2	C25_2	118.6(3)
C11_1	Si6_1	C10_1	109.7(2)	C29_2	C24_2	P2_2	123.4(3)
C11_1	Si6_1	C12_1	108.2(3)	C25_2	C24_2	P2_2	118.0(3)
C10_1	Si6_1	C12_1	108.1(2)	C26_2	C25_2	C24_2	120.8(4)
C11_1	Si6_1	Si5_1	109.57(16)	C25_2	C26_2	C27_2	120.0(4)
C10_1	Si6_1	Si5_1	111.22(17)	C28_2	C27_2	C26_2	119.6(4)
C12_1	Si6_1	Si5_1	109.98(19)	C27_2	C28_2	C29_2	121.1(4)
C13_1	Si7_1	C15_1	108.6(3)	C28_2	C29_2	C24_2	119.8(4)
C13_1	Si7_1	C14_1	108.0(3)	C35_2	C30_2	C31_2	117.5(3)
C15_1	Si7_1	C14_1	108.1(3)	C35_2	C30_2	P2_2	123.0(3)
C13_1	Si7_1	Si5_1	110.40(18)	C31_2	C30_2	P2_2	119.4(3)
C15_1	Si7_1	Si5_1	112.23(17)	C30_2	C31_2	C32_2	121.4(4)
C14_1	Si7_1	Si5_1	109.39(16)	C33_2	C32_2	C31_2	119.7(4)
C16_1	Si8_1	C17_1	109.8(2)	C34_2	C33_2	C32_2	120.0(4)
C16_1	Si8_1	C18_1	109.1(2)	C33_2	C34_2	C35_2	119.9(4)
C17_1	Si8_1	C18_1	108.2(2)	C30_2	C35_2	C34_2	121.5(4)
C16_1	Si8_1	Si5_1	107.64(15)	C41_2	C36_2	C37_2	119.4(3)
C17_1	Si8_1	Si5_1	111.20(14)	C41_2	C36_2	P2_2	116.8(2)
C18_1	Si8_1	Si5_1	110.92(15)	C37_2	C36_2	P2_2	123.8(3)
Si11_1	Si9_1	Si10_1	110.94(5)	C38_2	C37_2	C36_2	119.9(3)
Si11_1	Si9_1	Si12_1	110.82(5)	C39_2	C38_2	C37_2	120.5(3)
Si10_1	Si9_1	Si12_1	111.28(5)	C38_2	C39_2	C40_2	119.8(3)
Si11_1	Si9_1	Ge3_1	105.89(4)	C39_2	C40_2	C41_2	120.4(3)
Si10_1	Si9_1	Ge3_1	111.56(5)	C40_2	C41_2	C36_2	119.9(3)
Si12_1	Si9_1	Ge3_1	106.12(4)	N1_2	C42_2	C43_2	178.0(4)
C19_1	Si10_1	C20_1	108.5(2)	N1_3	C1_3	C2_3	178.9(7)
C19_1	Si10_1	C21_1	108.7(2)	N2_3	C3_3	C4_3	179.5(9)
C20_1	Si10_1	C21_1	108.6(2)				

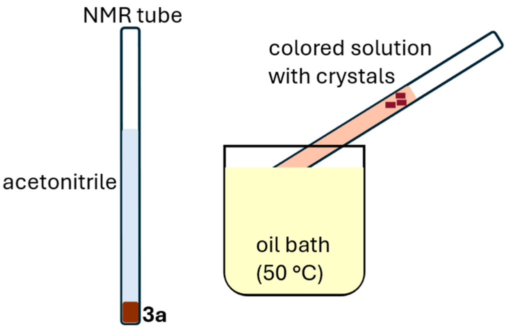

Figure S21. Setup for the crystallization of **3b** in a sealed NMR tube.
